# The Transcriptional Response to Oxidative Stress during Vertebrate Development: Effects of *tert*-Butylhydroquinone and 2,3,7,8-Tetrachlorodibenzo-*p*-Dioxin

**DOI:** 10.1371/journal.pone.0113158

**Published:** 2014-11-17

**Authors:** Mark E. Hahn, Andrew G. McArthur, Sibel I. Karchner, Diana G. Franks, Matthew J. Jenny, Alicia R. Timme-Laragy, John J. Stegeman, Bruce R. Woodin, Michael J. Cipriano, Elwood Linney

**Affiliations:** 1 Biology Department, Woods Hole Oceanographic Institution, Woods Hole, Massachusetts, United States of America; 2 Josephine Bay Paul Center for Comparative Molecular Biology and Evolution, Marine Biological Laboratory, Woods Hole, Massachusetts, United States of America; 3 Department of Molecular Genetics and Microbiology, Duke University Medical Center, Durham, North Carolina, United States of America; Oregon State University, United States of America

## Abstract

Oxidative stress is an important mechanism of chemical toxicity, contributing to teratogenesis and to cardiovascular and neurodegenerative diseases. Developing animals may be especially sensitive to chemicals causing oxidative stress. The developmental expression and inducibility of anti-oxidant defenses through activation of NF-E2-related factor 2 (NRF2) affect susceptibility to oxidants, but the embryonic response to oxidants is not well understood. To assess the response to chemically mediated oxidative stress and how it may vary during development, zebrafish embryos, eleutheroembryos, or larvae at 1, 2, 3, 4, 5, and 6 days post fertilization (dpf) were exposed to DMSO (0.1%), *tert*-butylhydroquinone (tBHQ; 10 µM) or 2,3,7,8-tetrachlorodibenzo-*p*-dioxin (TCDD; 2 nM) for 6 hr. Transcript abundance was assessed by real-time qRT-PCR and microarray. qRT-PCR showed strong (4- to 5-fold) induction of *gstp1* by tBHQ as early as 1 dpf. tBHQ also induced *gclc* (2 dpf), but not *sod1, nqo1, or cyp1a*. TCDD induced *cyp1a* but none of the other genes. Microarray analysis showed that 1477 probes were significantly different among the DMSO-, tBHQ-, and TCDD-treated eleutheroembryos at 4 dpf. There was substantial overlap between genes induced in developing zebrafish and a set of marker genes induced by oxidative stress in mammals. Genes induced by tBHQ in 4-dpf zebrafish included those involved in glutathione synthesis and utilization, signal transduction, and DNA damage/stress response. The strong induction of *hsp70* determined by microarray was confirmed by qRT-PCR and by use of transgenic zebrafish expressing enhanced green fluorescent protein (EGFP) under control of the hsp70 promoter. Genes strongly down-regulated by tBHQ included *mitfa*, providing a molecular explanation for the loss of pigmentation in tBHQ-exposed embryos. These data show that zebrafish embryos are responsive to oxidative stress as early as 1 dpf, that responsiveness varies with development in a gene-specific manner, and that the oxidative stress response is substantially conserved in vertebrate animals.

## Introduction

Oxidative stress occurs when redox signaling and control are disrupted, either through generation of non-physiological levels of reactive oxygen species (ROS) or by alterations in the regulation of key thiol/disulfide couples [1]. Oxidative stress has been cited as a causative or contributing factor in a variety of human conditions linked to environmental exposures, ranging from chemical teratogenesis to cardiovascular and neurodegenerative diseases [2]–[6]. Redox-mediated signaling is thought to be important for cellular differentiation during embryonic development [7], [8] and developing embryonic stages may be especially sensitive to the disrupted redox and sulfhydryl balance that characterizes oxidative stress [9]–[12]. Oxidative damage has been implicated in the mechanism of action of several developmental toxicants, including known human teratogens (e.g. thalidomide, phenytoin, ethanol), environmental contaminants (e.g. 2,3,7,8-tetrachlorodibenzo-p-dioxin (TCDD), benzo[a]pyrene), and nanomaterials [9], [13], [14], as well as in the etiology of congenital malformations associated with diabetic embryopathy [15], [16].

The constitutive (basal) expression and inducibility of anti-oxidant defenses are known to affect the susceptibility of adult tissues and cells to effects of oxidative stress [17], [18], and are likely to be important determinants of susceptibility at early life stages as well [19], [20]. In adult animals, oxidant and pro-oxidant chemicals elicit an *oxidative stress response* (OSR), which involves the increased expression of genes whose products act to mitigate the oxidant challenge. Oxidants, electrophiles, sulfhydryl-reactive agents, and some phenolic anti-oxidants initiate this response by activating NF-E2-related factor 2 (NRF2 [NFE2L2]) and related cap'n'collar (CNC)-basic-leucine zipper (bZIP) family proteins. (For nomenclature conventions, please see footnote 3 of reference [21].) NRF2 is normally found in the cytoplasm, where an interaction with Kelch-like-ECH-associated protein (KEAP1) targets it for rapid proteasomal degradation [22]. Oxidative stress disrupts the interaction between NRF2 and KEAP1, after which NRF2 enters the nucleus and forms a heterodimer with one of several small Maf proteins; NRF2-Maf dimers bind to anti-oxidant response elements (ARE) and activate transcription of genes such as glutathione S-transferases (GST), NAD(P)H-quinone oxidoreductase (NQO1), glutamate-cysteine ligase (catalytic subunit; GCLC), heme oxygenase (HMOX), and superoxide dismutase (SOD) [18], [23], [24].

Despite the demonstrated importance of the OSR in adults, the ability of vertebrate early life stages to respond to oxidative insult is not well understood. How does the sensitivity of developing vertebrates to oxidative stress vary with developmental stage? Are the patterns of induced or repressed gene expression stage-specific? What transcription factors are involved in regulating the OSR in embryos?

To begin to address these questions and elucidate the fundamental mechanisms by which vertebrates respond to oxidative stress during development, we have initiated studies to identify the core set of genes and the transcription factors involved in the OSR in developing zebrafish. The zebrafish (*Danio rerio*) is an established model in developmental biology that has emerged also as a valuable *in vivo* system in which to examine mechanisms of toxicity in developing animals and to screen chemicals for developmental toxicity [3], [25]–[30]. Thus, we used zebrafish as a model system in which to explore the mechanisms by which vertebrate early life stages respond to oxidative stress [31]–[37].

Previous studies carried out in zebrafish or zebrafish cells have established the evolutionary conservation of the OSR, including the roles of Nrf2 [33], [38], [39], Keap1 [33], [39], [40], and AREs [32], [41]–[43]. Because of a whole-genome duplication that occurred in fish after the divergence of the fish and mammalian lineages, zebrafish and other teleost fish often possess duplicates (paralogs) of single mammalian genes, and the duplicates have often partitioned the subfunctions of their mammalian ortholog [44], [45]. Consistent with this, zebrafish possess two KEAP1 paralogs (Keap1a and Keap1b) with distinct functions in regulating the OSR [39], [40], as well as paralogs of NRF1 (Nrf1a and Nrf1b) and NRF2 (Nrf2a and Nrf2b) [21], [33], [46]. Such studies indicate that novel insights may be obtained by studying the OSR in zebrafish.

Two fundamental questions concerning the OSR and its regulation in zebrafish remain unanswered. First, the ontogeny of the OSR in zebrafish embryonic and larval stages is not well understood. Kobayashi *et al.* [33], [39] reported that *tert*-butylhydroquinone (tBHQ) can induce an OSR in zebrafish larvae (4 to 7-dpf), but that embryos at 8–24 hpf were incapable of mounting an acute OSR (as measured by induction of *gstp1*). Timme-Laragy et al. [38] observed an OSR (induction of *gstp1*, *gpx1*, and *gclc*) in zebrafish embryos exposed to *tert*-butylhydroperoxide or ß-naphthoflavone for 24 hr beginning at 24-hpf. These reports suggest that there are stage- or chemical-specific differences in responsiveness to oxidative stress, highlighting the need for a more systematic assessment of the basal (constitutive) and oxidant-inducible expression of OSR genes during development. Second, the set of oxidant-responsive genes in adult mammals or mammalian cell culture has been studied extensively (e.g. [47], [48]), but the overlap between the OSR in zebrafish and mammals, and the identity of genes that respond to oxidative stress in developing vertebrates, are not yet well understood.

**Figure 1:**
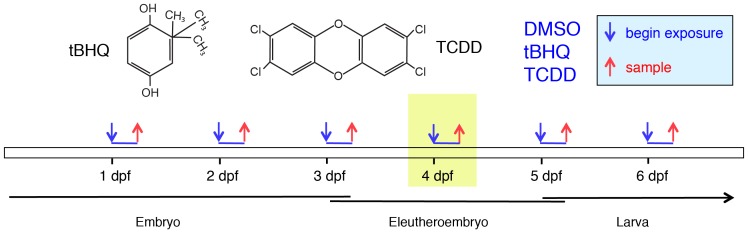
Exposure and sampling regime. Zebrafish embryos, eleutheroembryos, or larvae were exposed to DMSO, tBHQ (10 µM), or TCDD (2 nM) at 1, 2, 3, 4, 5, or 6-dpf for 6 hr (4 groups per compound per time point), and sampled immediately followed exposure for isolation of RNA as described in *Materials and Methods*. The yellow shading indicates the time-point chosen for the microarray analysis. Phases of zebrafish development are not absolute but are categorized here as embryos (1, 2, and 3-dpf), eleutheroembryos (4 and 5-dpf), and larvae (6-dpf) following the nomenclature of others [28], [49]).

Here we report the results of studies using expression profiling to assess the nature of the response of developing zebrafish to chemically mediated oxidative stress. We conducted experiments in which gene expression was measured in zebrafish exposed to tBHQ at various times during development, including embryos (1, 2, and 3-dpf), eleutheroembryos (4 and 5-dpf), and larvae (6-dpf) (developmental phases according to [28], [49]; Fig. 1). tBHQ is widely used as a prototypical mono-functional inducer of the OSR [23]. For comparison and to differentiate between NRF- and AHR-mediated responses, we also exposed developing zebrafish to TCDD, which is well known to cause altered gene expression through activation of AHRs in zebrafish embryos [50], [51], and has been suggested to cause embryotoxicity at least in part by causing oxidative stress [52]. The results identify both similarities and differences between the OSR in developing zebrafish as compared with that elicited by tBHQ in previous studies in adult mammals or mammalian cells. In addition, we identify a link between a specific change in gene expression (repression of *mitfa*) and a phenotypic response to tBHQ (loss of pigmentation) and we describe the potential use of an existing transgenic line of zebrafish [53] to further investigate the temporal and spatial regulation of the anti-oxidant response in developing vertebrates.

## Results

### Expression and induction of oxidative stress response genes in zebrafish embryos

To assess the ability of embryos to mount a response to oxidative stress, we performed an experiment in which separate groups of embryos, eleutheroembryos, or larvae at 1, 2, 3, 4, 5, and 6-dpf were exposed for 6 hr to DMSO, tBHQ (10 µM), or TCDD (2 nM) and gene expression was measured by qRT-PCR and microarray. The goal of this experiment was to assess the acute response (6 hr) to chemical treatment occurring at different stages of development (**Fig. 1**), rather than secondary changes that might occur after a longer exposure time.

**Figure 2:**
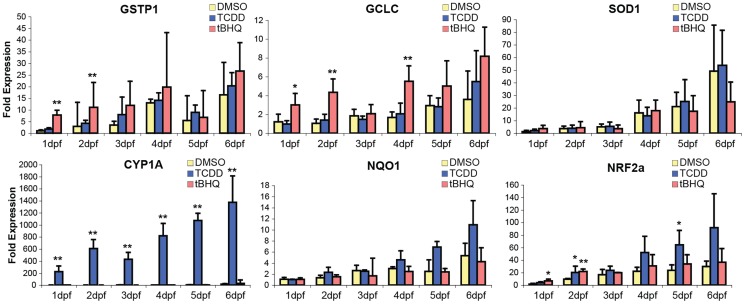
Changes in gene expression in developing zebrafish following exposure to TCDD (2 nM) or tBHQ (10 µM) in 0.1% DMSO. Embryos, eleutheroembryos, or larvae at 1, 2, 3, 4, 5, or 6-dpf were exposed to each chemical for 6 hours, after which they were frozen for RNA isolation and analysis of gene expression. Expression of *gstp1, gclc, sod1, cyp1a1, nqo1, and nrf2a* genes were measured by qRT-PCR. Primers targeting *gstp1* may also measure the closely related *gstp2*. Values represent mean±SE of 4 biological replicates. *statistical significance at p<0.05 (Dunnett's test).

Targeted analysis of gene expression by qRT-PCR showed that tBHQ caused strong (4- to 8-fold) induction of *gstp1* in 1- and 2-dpf embryos, but not at later stages (**Fig. 2**). tBHQ also induced *gclc* (3- to 4-fold; 1-, 2-, and 4-dpf) and *nrf2a* (1- and 2-dpf), but not *sod1*, *nqo1*, or *cyp1a*. TCDD induced *cyp1a* at all time points (100- to 600-fold) and *nrf2a* at 2- and 5-dpf (2- to 3-fold), but did not significantly alter the expression of the other genes examined, under these exposure conditions. The induction of *nrf2a* expression by TCDD is consistent with recent studies showing regulation of *NRF2* expression by the AHR in mammals [54], [55]. Overall, the data demonstrated that some classical OSR genes (*gstp1*, *gclc*) were responsive to tBHQ-induced oxidative stress in embryos as early as 1- and 2-dpf, while others (*sod1* and *nqo1*) were not inducible by a 6-hr exposure to this concentration of tBHQ (10 µM) at any of the developmental stages examined here.

To more comprehensively assess the set of oxidant-responsive genes in zebrafish embryos, we examined a subset of the RNA samples by microarray using the Agilent 22k zebrafish array. We analyzed all of the 4-dpf samples, which included four biological replicates each for DMSO-, tBHQ- and TCDD-treated embryos. The 4-dpf time was chosen for gene expression profiling because Kobayashi *et al.* [33], [39] had reported a robust induction of several oxidant-responsive genes by tBHQ in zebrafish larvae at this stage. Probes indicating significantly different relative transcript abundance among the DMSO, TCDD, and tBHQ treatments were determined by ANOVA, with the false discovery rate (FDR) set to 5%. Overall, 1477 probes exhibited significant differences in expression among the three exposure groups. For each of the probes we calculated fold-change in gene expression for TCDD- and tBHQ-treated embryos versus DMSO-treated embryos (**Table S2**).

FatiGO+ [56] was used to examine enrichment of Gene Ontology (GO) terms in the set of significant probes relative to the entire microarray probe set. GO terms enriched in the set of probes detecting transcripts that were up-regulated in response to tBHQ included “glutathione metabolic process”, “response to temperature stimulus”, “protein dimerization activity”, and several categories involving ester hydrolases and protein phosphatases (**Table S3**). Down-regulation of transcript abundance by tBHQ was associated with enrichment of GO terms for “negative regulation of cellular process”, “regulation of transcription”, “transcription”, and several categories of proteases (**Table S3**).

(The ability to perform GO analyses was limited by the incomplete annotation of probes on the Agilent array and by incomplete GO annotation of the zebrafish genome. Thus, when interpreting the FatiGO+ results it is important to consider the current status of zebrafish genome annotation. FatiGO+ relies on the assignment of GO terms within the Ensembl annotation of the zebrafish genome. However, many Agilent microarray probes have yet to be assigned to transcripts or genes at Ensembl and are thus not assigned GO terms. Others are assigned to transcripts or genes with preliminary annotation and GO term assignment only and are thus relatively uninformative, restricting FatiGO+ to detection of broad GO terms only (e.g. catabolic process, transcription, protein dimerization activity).)

**Figure 3:**
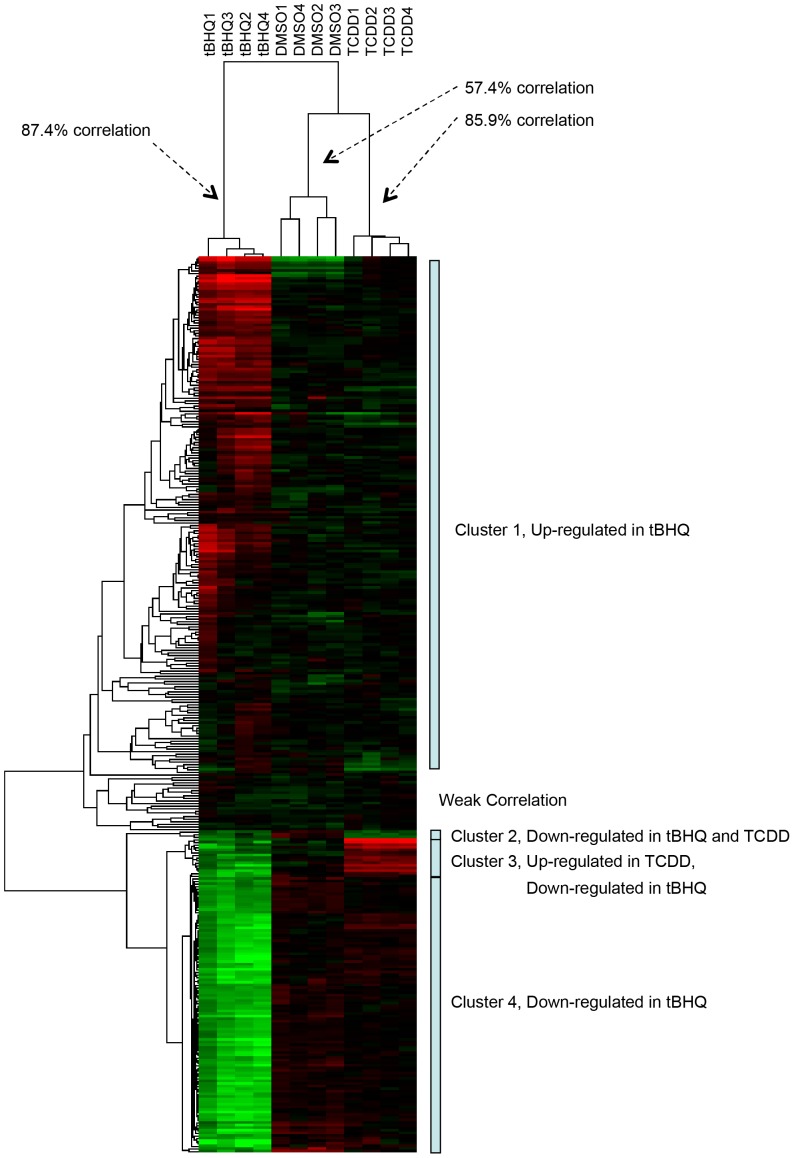
Heat map illustrating changes in gene expression in zebrafish eleutheroembryos following exposure to DMSO (0.1%), tBHQ (10 µM), or TCDD (2 nM). Eleutheroembryos at 4-dpf were exposed to each chemical for 6 hours, after which RNA was isolated, cRNA prepared and hybridized against a universal reference cRNA on Agilent 22k zebrafish arrays. There were 4 biological replicates, each a pool of 30 eleutheroembryos. The probes shown exhibited statistically significant differences among groups and at least 2-fold change (tBHQ vs. DMSO or TCDD vs. DMSO).

To prioritize the 1477 genes with significant changes for further study, we chose to focus only on those genes exhibiting ≥2-fold change in expression (up or down) for either TCDD or tBHQ, of which there were 345 (**Table S4**). This additional filter yielded 220 probes that were both significant in the statistical analysis and ≥2-fold up-regulated by tBHQ, and 109 that were significant and ≥2-fold down-regulated by tBHQ. For TCDD, 17 of the significant probes were ≥2-fold up-regulated and 8 were ≥2-fold down-regulated. There was very little overlap in probes responsive to tBHQ and TCDD (**Fig. 3**). At the ≥2-fold level, four probes (two *hsp70* probes, *dnajb1*, *hsp90a2*) were up-regulated by both tBHQ and TCDD and two probes were down-regulated by both compounds; one probe (*foxq1b*) was up-regulated in response to TCDD but down-regulated in response to tBHQ, while two probes were down-regulated in response to TCDD but up-regulated in response to tBHQ. After all annotation efforts, 14 of the 345 probes (4.1%) did not have a significant match to known genes or expressed sequence tags (ESTs).

### Genes altered in response to tBHQ: Conserved vertebrate response to oxidative stress

One goal of this study was to compare the transcriptional response of developing zebrafish to a model oxidant (tBHQ) with the response that has been documented previously in adult mammals or mammalian cell lines. Any discrepancies might suggest either fish-mammal or embryo/larval-adult differences in the nature of the oxidative stress response. We therefore compared the set of genes induced by tBHQ in our experiment (**Table S2**) to a set of 18 “biological marker” genes that are induced by oxidative stress in mammals and mammalian cells through activation of the NRF2-ARE pathway [47]. Of the 18 markers, homologs of ten (56%) were up-regulated in 4-dpf zebrafish eleutheroembryos, five were unchanged, and three were not present on the array (**Table 1**). Several of the marker genes occur as duplicates in zebrafish; for four of these (*hsp90*, *dnajb1*, thioredoxin, ferritin heavy polypeptide), only one of the paralogs was induced by tBHQ.

**Table 1:** Zebrafish vs mammalian response to tBHQ.

Marker gene in mammals	Zebrafish (co)-orthologs	# probes	tBHQ/DMSO
HSP70 1A, 1B, 6	**hsp70 (Chr.3)**	2	**52.3±13.8**
	**hsp70l (Chr.8)**	1	**15.5±4.50**
HSP70 9B	hspa9 (Chr.14)	2	1.19±0.22
HSP90 1 alpha	hsp90a (Chr.20)	3	1.18±0.32
	**hsp90a2 (Chr.20)**	2	**10.4±1.80**
DnaJ (Hsp40) B1	dnajb1a (Chr.3)	3	1.39±0.07
	**dnajb1b (Chr.1)**	1	**10.48±2.33**
Heme oxygenase-1 (HMOX1)		0	NA
NAD(P)H quinone oxidoreductase-1 (NQO1)	nqo1	1	0.66±0.08
Glutamate-cysteine ligase, modifier subunit	**gclm**	1	**4.11±0.18**
Thioredoxin (TXN)	**txn1 (Chr.7)**	2	**5.47±0.92**
	txn2 (Chr.1)	2	0.83±0.21
Thioredoxin reductase-1	**txnrd1**	1	**1.56±0.14**
Malic enzyme 1		0	NA
Glutathione reductase	**glutathione reductase 1**	2	**3.79+−0.68**
Ferritin, heavy polypeptide-1	fth1 (Chr.7)	5	0.98±0.04
	**ferritin-like (Chr.3)**	2	**6.81±1.14**
	ferritin-like (Chr.25)	1	0.66±0.06
Ferritin light polypeptide	ferritin L	3	0.89±0.10
Carbonyl reductase-1	cbr1	2	1.09±0.27
Phosphogluconate dehydrogenase	**pgd**	2	**2.46±0.40**
Sequestosome-1	**sqstm1**	1	**7.57±1.56**
Ubiquitin thioesterase	usp4	2	1.00±0.08
Spermidine/spermine N1-acetyltransferase		0	NA

Set of 20 candidate genes based on the multiple data sets of genes responding to oxidative stress in mammalian cells, compiled by Johnson et al, as listed in Table 2 of Li et al *Physiol Genomics* 21: 43–58, 2005. The 20 mammalian candidate genes have been collapsed into 18 sets based on orthologous or co-orthologous relationships with zebrafish genes. Of 18 genes, 10 have at least one co-ortholog induced by tBHQ in 4-dpf zebrafish (indicated in **bold** type), 5 are not induced, and 3 are not found on array.

To further assess the nature of the eleutheroembryo response to tBHQ, we looked for altered expression of genes involved in the synthesis and utilization of glutathione (GSH) and other sulfhydryl-reactive anti-oxidants (thioredoxin, peroxiredoxin) and their regulation (**Table 2**). Among the induced genes, those associated with GSH synthesis included *gclc*, *gclm*, and glutathione synthase (*gss*). Induced genes associated with GSH utilization included GSH reductase (*gr1*), gamma-glutamyl transferase (*ggt1a*), GST omega, and a microsomal GST. Thioredoxin (*txn1*) and cystathionine beta-synthase (cbsb), which are involved in cysteine disulfide reduction and cysteine synthesis via transsulfuration, respectively, also were induced by tBHQ.

**Table 2:** Changes in gene expression in 4-dpf zebrafish eleutheroembryos exposed to tBHQ or TCDD for 6 hr, relative to DMSO-treated eleutheroembryos: Selected genes involved in phase I & phase II biotransformation, GSH synthesis and utilization, oxidative stress response.

Probe number	Gene	tBHQ	TCDD
		mean ± SE	mean ± SE
A_15_P110246	catalase	0.90+−0.14	0.85+−0.03
A_15_P117964	superoxide dismutase 1, soluble (sod1)	1.12+−0.21	0.80+−0.07
A_15_P110713	superoxide dismutase 2, mitochondrial (sod2)	0.81+−0.03	0.83+−0.04
A_15_P108217	NAD(P)H dehydrogenase, quinone 1 (nqo1)	0.66+−0.08	1.19+−0.05
A_15_P107652	**prostaglandin endoperoxide synthase 2 (ptgs)**	**5.89+−0.48**	0.61+−0.06
A_15_P100578	**cytochrome P4501A (cyp1a)**	0.96+−0.09	**71.59+−6.09**
A_15_P102530	**UDP glycosyltransferase (ugt1b5)**	0.66+−0.15	**4.03+−0.48**
A_15_P101728	epoxide hydrolase 1, like (ephx1l)	1.03+−0.25	1.20+−0.16
A_15_P100082	**glutamate-cysteine ligase c (gclc)**	**2.21+−0.12**	0.97+−0.04
A_15_P112437	**glutamate-cysteine ligase m (gclm)**	**4.11+−0.18**	1.33+−0.09
A_15_P102996	**g-glutamyl transferase (ggt1a)**	**7.31+−2.65**	0.84+−0.07
A_15_P109364	**glutathione reductase (gr1)**	**3.79+−0.68**	1.12+−0.04
A_15_P120619	glutathione peroxidase 4b (gpx)	0.81+−0.06	0.89+−0.06
A_15_P118489	**glutathione synthase (gss)**	**2.19+−0.26**	1.17+−0.11
A_15_P112576	**cystathionine beta-synthase (cbsb)**	**2.07+−0.34**	0.95+−0.07
A_15_P111318	glutathione S-transferase, alpha-like (gstal)	1.50+−0.27	0.68+−0.04
A_15_P118878	glutathione S-transferase, mu (gstm)	1.14+−0.18	0.87+−0.03
A_15_P107422	glutathione S-transferase, pi (gstp1)	2.89+−0.65	1.50+−0.08
A_15_P111132	**microsomal gst 3**	**2.22+−0.12**	0.95+−0.08
A_15_P109839	**glutathione S-transferase, omega**	**5.25+−1.30**	0.77+−0.04
A_15_P103106	**Thioredoxin** (txn1)	**5.47+−0.92**	1.04+−0.08
A_15_P107290	similar to vertebrate heme oxygenase decycling 2 (hmox2)	0.97+−0.11	1.03+−0.13
A_15_P115043	metallothionein (mt)	1.07+−0.13	0.92+−0.08
A_15_P105247	metallothionein 2 (mt2)	1.92+−0.19	0.95+−0.09

Microarray probes with significant (p<0.05, ANOVA with 5% FDR correction) change for tBHQ or TCDD relative to DMSO are in **bold type**. Data represent mean ± standard error of ratio: treated/DMSO.

There was strong induction of *keap1a* and several genes encoding small maf proteins (*mafk, maff, mafb*), which are involved in the NRF2 signaling pathway. In contrast, four of the six predicted NRF-family transcription factors (*nfe2*, *nrf1a*, *nrf1b*, and *nrf2a*) were represented on the array, but none showed significantly altered expression in tBHQ-exposed eleutheroembryos.

Another set of genes of interest was those involved in general stress responses. The gene induced most highly by tBHQ (more than 50-fold) was *hsp70*, which was represented by several probes on the array (**Table 3**). A *v-fos* homolog was also strongly induced (47-fold); there were also substantial increases in expression of *junb* (8.7-fold) and other jun-related transcripts. Other induced genes included an hsp90 isoform (*hsp90-alpha2*) and *hsp40/dnaJ*. The latter joined *atf3* and *gadd45* (several forms) as induced genes associated with a DNA-damage response.

**Table 3:** Changes in gene expression in 4-dpf zebrafish eleutheroembryos exposed to tBHQ or TCDD for 6 hr, relative to DMSO-treated eleutheroembryos: Other stress-responsive genes and transcription factors.

Probe number	Gene	tBHQ	TCDD
	*stress response*		
A_15_P110618	**heat shock cognate 70-kd protein (hsp70)**	**52.28+−13.8**	**3.87+−0.77**
A_15_P107601	**activating transcription factor 3 (atf3)**	**19.05+−2.71**	1.07+−0.05
A_15_P108778	**DnaJ (Hsp40) homolog, subfamily B, member 1 (dnajb1)**	**10.48+−2.33**	**2.17+−0.18**
A_15_P118357	**heat shock protein 90-alpha 2 (hsp90a2)**	**10.44+−1.79**	1.84+−0.12
A_15_P105326	**growth arrest and DNA-damage-inducible, beta (gadd45b)**	**10.20+−2.87**	1.10+−0.10
A_15_P105778	**insulin-like growth factor binding protein 1 (igfbp1)**	**8.38+−1.06**	0.89+−0.15
A_15_P114842	**hepcidin antimicrobial peptide 1 (hamp1)**	**8.32+−3.23**	0.45+−0.14
A_15_P102163	**hypoxia induced gene 1 (hig1)**	**7.65+−1.11**	1.16+−0.06
A_15_P119378	**sequestosome 1 (sqstm1)**	**7.57+−1.56**	1.26+−0.07
A_15_P113284	**growth arrest and DNA-damage-inducible, alpha like (gadd45al)**	**3.41+−0.54**	1.41+−0.06
	*Transcription factors/signal transduction*		
A_15_P101481	**v-fos FBJ murine osteosarcoma viral oncogene homolog (fos)**	**46.78+−9.17**	1.90+−0.11
A_15_P102446	**similar to jun dimerization protein**	**9.21+−1.00**	0.96+−0.05
A_15_P117758	**jun B (junb)**	**8.71+−0.81**	1.10+−0.09
A_15_P101236	**v-jun sarcoma virus 17 oncogene homolog (avian) (jun)**	**2.69+−0.23**	0.89+−0.07
A_15_P119415	**suppressor of cytokine signaling 3 (socs3)**	**6.45+−1.62**	1.21+−0.13
A_15_P113920	**CCAAT/enhancer binding protein (C/EBP), beta (cebpb)**	**6.24+−1.28**	0.93+−0.04
A_15_P118944	**sprouty (Drosophila) homolog 4 (spry4)**	**5.05+−0.85**	1.19+−0.03
A_15_P104781	**v-maf musculoaponeurotic fibrosarcoma oncogene homolog K (avian) (mafk)**	**4.08+−0.60**	1.14+−0.12
A_15_P102698	**v-maf musculoaponeurotic fibrosarcoma oncogene homolog f (avian) (maff)**	**3.06+−0.29**	1.23+−0.13
A_15_P119243	**v-maf musculoaponeurotic fibrosarcoma oncogene family, protein B (avian) (mafb)**	**2.04+−0.19**	1.01+−0.13
A_15_P101990	**SRY-box containing gene 9a (sox9a)**	**2.44+−0.29**	0.89+−0.03
A_15_P109504	nuclear factor (erythroid-derived 2)-like 2 (nfe2l2; nrf2a)	1.30+−0.04	1.19+−0.08
A_15_P110831	nuclear factor, erythroid derived 2,-like 1 (nfe2l1; nrf1a))	0.63+−0.21	0.72+−0.19
A_15_P116909	nuclear factor, erythroid derived 2,-like 1 (nfe2l1; nrf1b)	2.38+−0.51	2.19+−1.05
A_15_P109440	nuclear factor, erythroid-derived 2 (nfe2)	0.63+−0.08	0.81+−0.15
A_15_P105139	kelch-like ECH-associated protein 1 (keap1)	3.55+−0.64	1.57+−0.23
A_15_P104554	jun B proto-oncogene, like (junbl)	11.42+−1.47	1.31+−0.03
A_15_P120520	aryl hydrocarbon receptor 1a (ahr1a)	0.64+−0.12	1.24+−0.13
A_15_P103538	**aryl hydrocarbon receptor 2 (ahr2)**	0.80+−0.04	**2.42+−0.24**
A_15_P105040	aryl hydrocarbon receptor nuclear translocator (arnt2)	1.38+−0.07	1.04+−0.04
A_15_P102120	**forkhead box Q1 (foxq1b)**	0.43+−0.06	**3.48+−0.13**

Microarray probes with significant (p<0.05, ANOVA with 5% FDR correction) change for tBHQ or TCDD relative to DMSO are in **bold type**. Data represent mean ± standard error of ratio: treated/DMSO.

#### cis-regulatory elements

Among the genes significantly up-regulated by tBHQ exposure in the microarray experiment, some contained a possible DRE (e.g. early growth response 2a, arrestin domain containing 2, angiotensinogen, dual specificity phosphatase 5, phosphogluconate dehydrogenase isoform 1), an NF-kappaB motif (e.g. kelch-like ECH-associated protein 1a, growth arrest and DNA-damage-inducible, beta), SP1 motif (arrestin domain containing 2, pyruvate dehydrogenase kinase 2, dual specificity phosphatase 5, hypoxia induced gene 1, mmp13), REL motif (kelch-like ECH-associated protein 1a, growth arrest and DNA-damage-inducible beta, myocyte enhancer factor 2d), the Mafb motif (e.g solute carrier family 25 member 43), and HIF1A::Arnt motif (e.g. heat shock cognate 70-like, solute carrier family 25 member 43).

For genes significantly down-regulated by tBHQ exposure, a number contained an SP1 motif (e.g. forkhead box D1-like, growth arrest-specific 1a, red-sensitive opsin-1, microphthalmia-associated transcription factor a, insulin-like growth factor 2a precursor) and a Mafb motif (rad and gem-related GTP-binding protein 1, aquaporin 3a, red-sensitive opsin-1).

#### Novel responses to tBHQ

In addition to the stress-responsive genes and those involved in GSH homeostasis and other adaptive responses to oxidative stress, there were several notable changes in expression of other genes, including some with important roles during development (**Table 3** **; Table S2**). For example, *sox9a*, which has roles in development of chondrocytes and the retina [57], [58], was induced 2.4-fold by tBHQ. The fibroblast growth factor inhibitor *sprouty4* was induced 5-fold. Transcripts for the iron-regulatory protein hepcidin (hepcidin anti-microbial peptide; *hamp1*) were induced 8.3-fold. Prostaglandin endoperoxide synthase 2a (*ptgs2a*; also known as *cox2*) was induced almost 6-fold by tBHQ. Hypoxia-induced gene (*hig1*) was induced 7-fold. Several members of the solute carrier family (*slc25a25*, *slc16a9a*, *slc16a3*, *slc1a4*, *slc25a43*, *slc16a6b* and *slc13a2*) were strongly induced, suggesting a general up-regulation of transport activity. Several dual-specificity phosphatases (*dusp5, dusp4, dusp1*) also were induced.

Although reports of the response to oxidative stress often emphasize the genes that are induced, oxidative stress also leads to decreases in the expression of some genes. In zebrafish eleutheroembryos exposed to tBHQ at 4-dpf, almost a third of the genes with significant and ≥2-fold changes were down-regulated, several strongly so (**Table S2**). A number of these suggest effects on the eye. For example, opsin 1 (*opn1lw1*), micropthalmia-associated transcription factor a (*mitfa*), and genes involved in retinoid homeostasis (lecithin retinol acyltransferase a, retinol binding protein 4) were among those strongly suppressed by tBHQ exposure at 4 dpf. Several genes with known developmental roles also were repressed by tBHQ. Examples include *foxq1b*, frizzled homolog 2, kruppel-like factor 2a, distal-less homeobox gene 3b, lunatic fringe, noggin1, and fibroblast growth factor 8 (**Table 4**).

**Table 4:** Genes down-regulated in 4-dpf zebrafish eleutheroembryos exposed to tBHQ for 6 hr, relative to DMSO-treated eleutheroembryos.

Probe number	Gene	tBHQ	TCDD
A_15_P115644	v-myc myelocytomatosis viral oncogene homolog 1, lung carcinoma derived (avian) a (mycl1a)	0.50+−0.04	0.89+−0.10
A_15_P108775	caspase 6, apoptosis-related cysteine peptidase, like 1 (casp6l1)	0.49+−0.04	0.79+−0.06
A_15_P121041	noggin 1 (nog1)	0.48+−0.06	0.70+−0.11
A_15_P108022	lunatic fringe homolog (lfng)	0.47+−0.08	0.82+−0.05
A_15_P105107	sulfotransferase family 2, cytosolic sulfotransferase 1 (sult2st1)	0.47+−0.06	1.27+−0.12
A_15_P112525	insulin-like growth factor 2a (igf2a)	0.46+−0.04	1.08+−0.07
A_15_P116827	protein phosphatase 1, regulatory (inhibitor) subunit 3B (ppp1r3b)	0.46+−0.03	0.74+−0.04
A_15_P112836	frizzled homolog 2 (fzd2)	0.45+−0.06	1.01+−0.09
A_15_P104846	aquaporin 3a (aqp3a)	0.45+−0.04	0.73+−0.05
A_15_P111028	chemokine (C-X-C motif) receptor 7b (cxcr7b)	0.44+−0.08	1.20+−0.09
A_15_P102120	forkhead box Q1 (foxq1b)	0.43+−0.06	3.48+−0.13
A_15_P108178	cytochrome P450, family 17, subfamily A, polypeptide 1 (cyp17a1)	0.43+−0.05	0.92+−0.08
A_15_P110067	progestin and adipoQ receptor family member Vb (paqr5b)	0.42+−0.03	0.90+−0.11
A_15_P106239	cyclin B1 (ccnb1)	0.42+−0.03	0.91+−0.08
A_15_P111351	cytochrome P450, family 17, subfamily A, polypeptide 1 (cyp17a1)	0.42+−0.01	0.77+−0.06
A_15_P117241	distal-less homeobox gene 3b (dlx3b)	0.41+−0.09	1.85+−0.05
A_15_P100844	Aldo/keto reductase homolog	0.41+−0.05	0.91+−0.01
A_15_P118093	fibroblast growth factor 8 a (fgf8a)	0.40+−0.07	0.79+−0.04
A_15_P114727	malate dehydrogenase 1b, NAD (soluble) (mdh1b)	0.39+−0.12	0.96+−0.10
A_15_P104273	caspase b (caspb)	0.37+−0.02	1.07+−0.11
A_15_P104141	retinol binding protein 4, plasma (rbp4)	0.37+−0.01	0.89+−0.07
A_15_P109617	cyclin B2 (ccnb2)	0.37+−0.01	0.76+−0.08
A_15_P106832	caspase b (caspb)	0.35+−0.09	1.15+−0.22
A_15_P111412	melanoregulin (zgc:91968)	0.35+−0.07	0.93+−0.03
A_15_P106085	sciellin (scel)	0.35+−0.03	0.78+−0.03
A_15_P109688	annexin A1b (anxa1b)	0.34+−0.02	0.89+−0.03
A_15_P111328	microphthalmia-associated transcription factor a (mitfa)	0.33+−0.06	0.99+−0.17
A_15_P108040	Kruppel-like factor 2a (klf2a)	0.33+−0.04	1.17+−0.06
A_15_P107334	v-maf musculoaponeurotic fibrosarcoma (avian) oncogene homolog (maf)	0.33+−0.04	0.74+−0.06
A_15_P101484	lecithin retinol acyltransferase a (phosphatidylcholine–retinol O-acyltransferase a) (lrata)	0.21+−0.04	0.63+−0.07
A_15_P119724	opsin 1 (cone pigments), long-wave-sensitive, 1 (opn1lw1)	0.17+−0.03	0.86+−0.22

All probes listed in this table had significant (p<0.05, ANOVA with 5% FDR correction) and >2-fold decrease in expression in tBHQ-treated relative to DMSO treated embryos. Data represent mean ± standard error of ratio: treated/DMSO. Only a subset of the down-regulated genes is shown; for complete set, see Tables S2 and S4.

#### Genes altered in response to TCDD

The number of genes affected by TCDD was small in comparison to the number regulated by tBHQ. Not surprisingly, *cyp1a* exhibited the greatest degree of induction (>70-fold), confirming the effectiveness of the exposure regimen. *hsp70* also was induced by TCDD, but the induction was modest (less than 4-fold) as compared to that caused by tBHQ (>50-fold). The forkhead box gene *foxq1b* was induced 3.5-fold by TCDD. Other TCDD-induced genes included *ugt1b5* and *ahr2* (**Tables 2** **and** **3** **; see also Tables S2 and S4**). For those genes responding to TCDD, we detected cis-regulatory elements in Ahr2 (ARE, Arnt::Ahr, Mofb, HIF1A::Arnt), CYP1A (ARE, Arnt::Ahr, HIF1A::Arnt, SP1), and 5′ nucleotidase, cytosolic II, like 1 (ARE, Arnt::Ahr, SP1).

#### Analysis of selected genes by qRT-PCR

**Figure 4:**
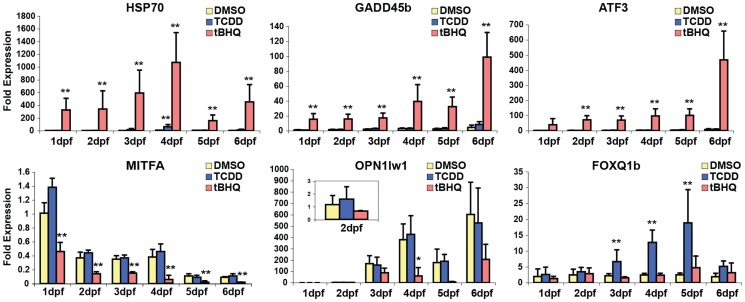
Changes in gene expression in developing zebrafish following exposure to TCDD (2 nM) or tBHQ (10 µM) in 0.1% DMSO. Embryos, eleutheroembryos, or larvae at 1, 2, 3, 4, 5, or 6-dpf were exposed to each chemical for 6 hours, after which they were frozen for RNA isolation and analysis of gene expression. Expression of *hsp70, gadd45b, atf3, mitfa, opn1lw1, and foxq1b* genes were measured by qRT-PCR. Values represent mean±SE of 4 biological replicates, each replicate a pool of 30 embryos. *statistical significance at p<0.05 (Dunnett's test).

To confirm the changes in gene expression measured by microarray and to further explore the timing of those changes with respect to zebrafish development, we considered the six genes originally chosen for targeted analysis by qRT-PCR (*gstp1*, *gclc*, *sod1*, *cyp1a*, *nqo1*, *nrf2a*; **Fig. 2**) and measured the expression of six additional genes, including both tBHQ up-regulated (*hsp70*, *gadd45*, *atf3*) and tBHQ down-regulated genes (*mitfa*, *opn1lw1*, *foxq1b*) (**Fig. 4**). There was excellent concordance between microarray data and day 4 qRT-PCR data (**Table S5**). Comparing the direction and statistical significance of the responses to tBHQ and TCDD for the twelve genes examined (i.e. 24 comparisons), there was agreement in all but one case: the decreased expression of *foxq1b* in tBHQ-exposed eleutheroembryos was not reproduced by qRT-PCR. Notably, *gstp1* showed a trend towards induction in both analyses (and was induced at other time points), but the high biological variation in the day 4 samples precluded statistical significance for both array data and qRT-PCR results. For all genes, the magnitude of change measured by qRT-PCR was as great or greater than that seen on microarray, in agreement with the well-known compression of fold-change values observed with array data [59]. Nevertheless, the confirmation of 23/24 microarray-detected changes by qRT-PCR supports the use of this platform for initial identification of tBHQ- and TCDD-induced changes in gene expression in developing zebrafish.

The qRT-PCR measurements also revealed varied developmental patterns of sensitivity to altered gene expression in response to tBHQ or TCDD during the first six days of development. Some genes were induced (*hsp70, gadd45b, atf3, cyp1a*) or repressed (*mitfa*) at all stages examined, whereas for other genes the altered expression occurred only at certain stages (*gstp, gclc, nrf2, opn1lw1, foxq1b*) or not at all (*nqo1, sod1*).

### Loss of pigmentation in embryos exposed to tBHQ linked to altered expression of *mitfa*

**Figure 5:**
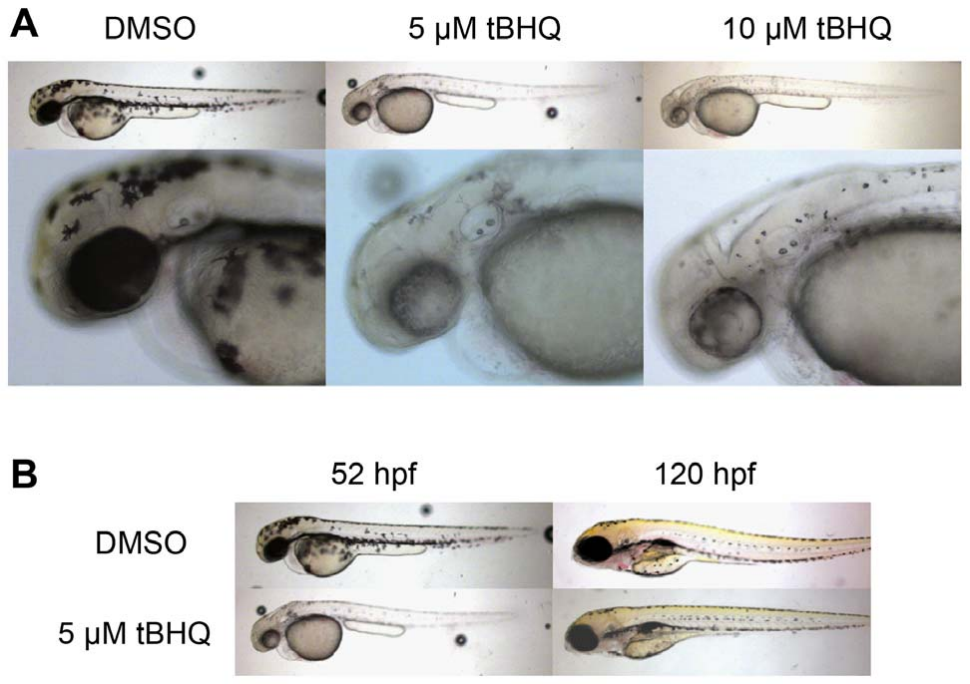
Phenotypic changes following tBHQ exposure. Zebrafish embryos were exposed to tBHQ (5 or 10 µM) or 0.1% DMSO from 32–47 hpf. **A.** At 52 hpf, randomly selected embryos were mounted in 3% methylcellulose and imaged as described in *Materials and Methods*. **B.** Embryos were subsequently maintained in 0.3× Danieau's water (without tBHQ) at until 120 hpf, when they were imaged again in order to assess recovery of pigmentation.

A recent report [60] noted briefly that zebrafish embryos exposed to tBHQ exhibited reduced pigmentation, an observation that was confirmed in our studies. To explore the timing and persistence of the effect of tBHQ on pigmentation, we exposed embryos to tBHQ (5 or 10 µM) from 32–47 hpf and examined pigmentation at 52 hpf. In DMSO-exposed embryos, melanophores could be observed in their normal positions near the otic vesicle, in the dorsal and ventral stripes, and around the yolk sac (**Fig. 5A**). In embryos exposed to tBHQ, melanophores in the head and trunk were present but were small and hypopigmented; melanophores were not apparent on the yolk sac. In addition, tBHQ-exposed embryos displayed a reduction in pigmentation in the retina. By 120-hpf (5-dpf), 3 days after tBHQ exposure had ended, there was partial recovery of pigmentation in the dorsal, lateral, and ventral stripes, in the yolk sac, and in the retina (**Fig. 5B**).

One of the genes most strongly repressed in tBHQ-treated eleutheroembryos (**Table 4** **;** **Fig. 4**) was *mitfa*, one of two zebrafish co-orthologs of the mammalian gene microphthalmia-associated transcription factor (*MITF*) [61]. The *mitfa* gene is defective in zebrafish *nacre* mutants, which lack melanophores and thus do not synthesize melanin other than in the retina [62]. The reduced expression of *mitfa* suggests a molecular explanation for the reduced pigmentation in tBHQ-treated embryos.

### Response of hsp70-EGFP transgenic zebrafish to tBHQ

There was a strong induction of *hsp70* at 4 dpf in response to tBHQ exposure (**Table 3** **,** **Fig. 4**) and studies in human cells have shown that *hsp70* expression can be regulated by NRF2 [63]. Together, these results suggested that the *hsp70* gene might serve as a useful marker for induction of the oxidative stress response during development.

**Figure 6:**
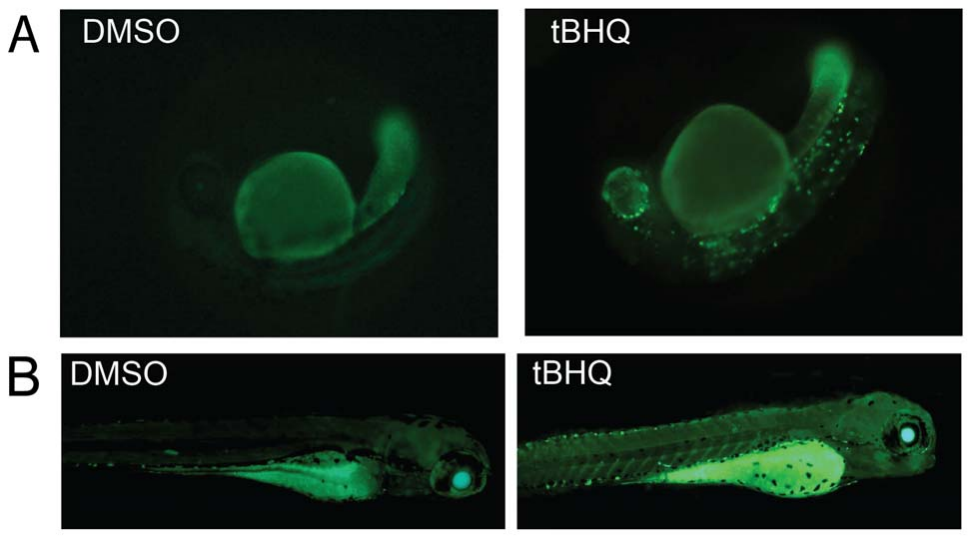
EGFP-HSP70 zebrafish exposed to tBHQ. **A.** Embryos from hsp70-EGFP transgenic zebrafish were exposed to DMSO (0.1%) or tBHQ (10 µM) for 4 hrs at 1-dpf. After exposure, embryos were washed and held for 4 additional hours prior to fluorescence microscopy for detecting EGFP expression. **B.** Another experiment was performed by exposing 4-dpf eleutheroembryos (n = 15) to DMSO (0.1%) or tBHQ (10 µM) for 4 hrs at 28°C. After exposure, eleutheroembryos were washed with 0.3× Danieau's and inspected 4 hrs post-exposure for EGFP expression by fluorescence microscopy.

Kuwada and coworkers [53] developed a transgenic line of zebrafish containing the gene for enhanced green-fluorescent protein (EGFP) under control of the zebrafish *hsp70* promoter. In unstressed fish, EGFP is expressed only in the lens of the eye [64]. To assess the response of the *hsp70-egfp* fish embryos to oxidative stress, we exposed them to tBHQ and looked for induction of EGFP. At 24 hpf, tBHQ (10 µM) induced EGFP expression in a restricted pattern of cells including the eye and in specific cells in the trunk (**Fig. 6A**). When *hsp70-EGFP* eleutheroembryos were exposed to tBHQ (10 µM) for 4 hr beginning at 96-hpf and examined 4 hr later (104 hpf, similar to the sampling point for our microarray studies), tBHQ induced widespread EGFP expression throughout the fish (**Fig. 6B**), consistent with the high level of induction of hsp70 measured by microarray and qRT-PCR.

## Discussion

The ability of embryos to protect themselves against oxidative damage is critical for maintaining developmental processes in the face of exposure to chemicals that are capable of disrupting redox balance and sulfhydryl metabolism. Despite the importance of such protective mechanisms, the ontogeny of constitutive and inducible antioxidant defenses in embryos is not well understood. Zebrafish serve as a valuable *in vivo* model to investigate the developmental regulation of the oxidative stress response. The results presented here and in our other recent papers [21], [35], [46] complement work done previously in this model [32], [33], [38]–[43], [60], [65], [66] by expanding the set of known NRF-related proteins potentially involved in regulating the OSR in zebrafish, determining the set of genes that are induced and repressed by a prototypical oxidant (tBHQ), and identifying a phenotype (loss of pigmentation) that is linked to a specific change in gene expression (decrease in *mitfa*).

### Gene expression profiles

An extensive OSR, including both increases and decreases in gene expression, occurred in zebrafish eleutheroembryos exposed to tBHQ for 6 hr at 4 dpf. We were particularly interested in the overlap between the OSR in zebrafish embryos and that described previously in mammalian systems (primarily adult tissues and cells). We also were interested in determining whether there were unexpected changes in gene expression that could represent novel responses in developing vertebrates. Importantly, there was concordance between microarray and qRT-PCR data (**Table S5**), validating the Agilent zebrafish microarray as a platform for evaluating expression profiles and for gene discovery in developing zebrafish exposed to oxidants. Computational searches for *cis*-regulatory elements involved in the observed responses to tBHQ and TCDD identified a number of possible regulatory mechanisms, particularly for tBHQ. The lack of hits for the NFE2L2 and NRF2 recognition sites was a product of the high false discovery rates for the mammalian *cis*-regulatory element models when used against the distant zebrafish genome and its divergent background nucleotide and dinucleotide frequencies. Experimental approaches such as chromatin immunoprecipitation sequencing (ChIP-Seq) may be better suited to resolve the targets of these regulatory proteins in the zebrafish.

#### Overlap in OSR between mammals and developing zebrafish

Many, but not all, of the known mammalian oxidant response genes were induced by tBHQ in zebrafish eleutheroembryos. More than half of the set of OSR marker genes identified by Johnson and colleagues [47] have zebrafish orthologs or co-orthologs that were induced by tBHQ in zebrafish at 4 dpf. Interestingly, in several cases in which there are two or more zebrafish co-orthologs of a mammalian gene (hsp90, dnajb1, thioredoxin, ferritin heavy polypeptide), only one of the zebrafish co-orthologs was induced (Table 1), suggesting that the zebrafish paralogs have divided the regulatory features of the mammalian ortholog such that only one form is inducible, a possible example of subfunction partitioning [45].

In addition to the 10 “biomarker” genes (Table 1), other well-known mammalian OSR genes were induced in zebrafish eleutheroembryos by tBHQ, including several involved in GSH and cysteine homeostasis: *gclc*, *gclm*, glutathione reductase (*gr1*), glutathione synthetase (*gss*), thioredoxin (*txn1*), cystathionine beta-synthase (*cbsb*) and two GSTs (an omega-class *gst* and a microsomal *gst*). We also saw strong induction of gamma-glutamyltransferase (*ggt1a*), which is involved in an extracellular GSH salvage pathway [67] and is important in protecting against oxidative DNA damage [68]. Other genes induced by tBHQ in our study that have also been reported to respond to oxidative stress in at least some mammalian systems include *atf3* and *dusp1* [47] (**Table 3**).

#### Overlap in OSR among studies in zebrafish embryonic stages and adults

Some of the OSR genes induced in our study (e.g. *txn1*, *gstp*, *gsto1 hsp70*, *dnaj*, *atf3*) were also shown to be induced by tBHQ in a study comparing the response of zebrafish embryos to several different toxicants, using a different and less complete microarray platform, published while this manuscript was in preparation [60]. Tanguay and colleagues [69] reported that fullerene (C_60_) caused an OSR in zebrafish embryos; genes induced in common by C_60_ [69] and tBHQ (this study) include *hsp70*, *gstp1*, *gclc*, and ferritin. There are no published studies showing effects of tBHQ on adult zebrafish. However, the response of adult zebrafish liver to arsenic (Na_2_HAsO_4_) exposure included genes associated with an OSR, including several that were also induced by tBHQ in our study (*hsp70*, *hsp90a*, ferritin, *gstp1*, *gsto1*, *txnrd1*, *txn*, *gadd45b*). Arsenic also induced hepatic expression of *sod2*, *gpx4b*, and *mt2*, which were not induced by tBHQ in embryos under the conditions examined in our experiment, demonstrating compound-, concentration-, tissue-, or stage-specific differences in the response to oxidants.

There are several other interesting differences in our data as compared to results obtained in other systems. Recent studies suggest that tBHQ is a weak AHR agonist in mammals [70], [71], but at the concentration used in our experiments (10 µM) tBHQ did not activate the AHR, as indicated by the absence of *cyp1a* induction in developing zebrafish at 1–6 dpf and by the negligible overlap between tBHQ- and TCDD-modulated gene sets.

Another notable aspect of our results is the genes not significantly affected by tBHQ exposure. For example, in contrast to what is observed in mammals [18], [47], there was no induction of *nqo1* or *sod1* by tBHQ as assessed either by microarray (in the 4-dpf eleutheroembryos) or by qRT-PCR (all time points). The lack of *nqo1* induction differs from the results of Kobayashi [33], who reported induction of this gene in 4-dpf zebrafish larvae exposed to 30 µM tBHQ for 6 hr. This could reflect a difference in the concentrations used in the two studies (10 µM vs. 30 µM). The induction of sod genes (*sod1* and *sod2*) in zebrafish embryos may be compound-specific, as suggested by the results of Timme-Laragy et al. [38], who observed induction of *sod1* and *sod2* after exposure of to a mixture of flavonoids but not after exposure to *tert*-butylhydroperoxide (at a concentration that induced other OSR genes such as *gclc* and *gstp1*). Despite the lack of induction of *nqo1* or *sod1* observed in our experiments, there may be other exposure conditions under which tBHQ might induce these genes.

#### Novel and notable responses

The microarray results also revealed novel changes in gene expression. We mention just a few of these to illustrate the apparent richness of the oxidative stress response in zebrafish eleutheroembryos. Hypoxia-induced gene (*hig1*) was induced 7-fold by tBHQ. This gene, first identified in fish exposed to hypoxia [72], is closely related to human hspc010 (hematopoietic stem-cell progenitor cell gene 10) [73]. tBHQ also strongly induced prostaglandin endoperoxide synthase 2 (*ptgs2; cox2*), dual-specificity phosphatases (*dusp5*, *dusp4*, and *dusp1*), insulin-like growth factor binding protein (*igfbp1*), and hepcidin anti-microbial peptide (*hamp*). These and other changes suggest the response to tBHQ in developing zebrafish involves, in addition to a classical OSR, transcriptional changes resembling an inflammatory response (*ptgs2*), response to hypoxia (*hig1*, *igfbp1*), and responses to maintain iron homeostasis (*hamp*, *ferritin*).

In tBHQ-treated eleutheroembryos at 4 dpf there was strong induction of genes associated with a DNA-damage response, such as *atf3* [74], *gadd45* (several forms), and *dnaJ/hsp40*, suggesting that oxidative DNA damage may be an early effect of this compound. We showed recently that the induction of *atf3* by tBHQ in zebrafish embryos was not controlled by Nrf2a or Nrf2b [21], consistent with the idea that at least some of these responses may be secondary to damage rather than a direct effect of tBHQ mediated through one of the Nrf2 proteins.

The results also revealed additional induced genes of interest. For example, several zebrafish members of the solute carrier family (e.g. *slc25a25, slc13a2, slc16a9a, slc16a3, slc1a4, slc16a6b*) were induced by tBHQ. Human *slc25a25* encodes a mitochondrial carrier that transports adenine nucleotides (ATP) across the inner mitochondrial membrane in exchange for phosphate [75]. Three monocarboxylate transporters (MCTs) (*slc16a6b*, *slc16a9a*, and *slc16a3*) were induced ∼3–5-fold; MCTs transport pyruvate, which can scavenge ROS [76]. *SLC* genes have not been generally recognized as part of the oxidative stress response. However, *SLC3A2* was recently shown to be induced in human umbilical vein endothelial cells exposed to lipid oxidation products derived from oxidized low-density lipoproteins [77], SLC3A2 and SLC1A4 (amino acid transporters) were induced in HepG2/C3A cells in response to cysteine deprivation [78], and SLC1A1 (a cysteine transporter) was induced by tBHQ and sulforaphane in rat glioma cells [79]. Although *slc3a2* and *slc1a1* were not among the *slc* genes induced in our experiment, overall the increase in *slc* gene expression suggests that induction of certain transport proteins might be an important part of the oxidative stress response, or a wider “integrated stress response” [80] in developing vertebrates. Induction of *slc* genes also occurs in response to other toxicants in zebrafish embryonic stages, but the patterns of *slc* gene expression may be toxicant-specific [60].

#### Implications for response to other oxidants

tBHQ is widely used as a prototypical mono-functional inducer of the OSR [23], but whether the results obtained here are representative of changes expected from exposure of developing zebrafish to other oxidant chemicals is not clear. The chemical specificity of the OSR has not been thoroughly investigated, especially during vertebrate development. Changes in gene expression (gene expression profiles) may vary according to the type or localization of oxidative stress, for example as caused by different types of pro-oxidant chemicals or other mechanisms of oxidative stress. Although some studies report similar expression profiles in response to different types of oxidative stress [81], [82], there is evidence for chemical-specific roles of Keap1 paralogs and mechanistically distinct classes of Nrf2 activators [39] and there are reports of distinct gene expression patterns from genetic versus chemical activation of NRF2 [83], [84]. Our recent studies show that Nrf2a and Nrf2b regulate distinct but partially overlapping sets of genes constitutively [21] and in response to tBHQ (manuscript in preparation). Thus, it will be important to determine how embryos respond to chemicals that generate oxidative stress via different mechanisms. Such studies are underway [85].

#### Response to TCDD

One goal of this work was to compare the transcriptional response of developing zebrafish to TCDD exposure with that caused by a model oxidant such as tBHQ. Oxidative stress has been implicated in effects of TCDD [86]–[88] including in embryonic stages [52], but after 6 hr of exposure to TCDD at 4 dpf we found no evidence for an oxidative stress response in the genes measured by microarray. Similarly, targeted analysis of gene expression by qRT-PCR at all six exposure times (days 1 through 6) showed no changes in classical OSR genes such as *gstp1*, *gclc*, or *sod1* (Fig. 2). Other investigators also found little evidence for an OSR in developing zebrafish exposed to TCDD [89]. Our experiments in whole animals (embryos, eleutheroembryos, and larvae) could have missed highly localized oxidative stress and resultant changes in gene expression. We did, however, observe induction of *gstp1* in whole embryos 48 hr after exposure to TCDD starting at 24 hpf (unpublished studies), suggesting that generation of oxidative stress or disruption of sulfhydryl balance may be delayed or may occur in embryos after prolonged exposure to this compound. Thus, although widespread oxidative stress does not appear to be part of the acute response to TCDD exposure, it may occur in specific cell types or be part of the response to longer-term exposure to TCDD.

One of the more interesting changes observed in TCDD-exposed embryos was the induction of a *foxq1* gene now called *foxq1b*. After our preliminary reports of these data [90], [91] and while this manuscript was in preparation, Planchart & Mattingly [92] reported the induction of a different *foxq1b* gene (now called *foxq1a*) by TCDD in zebrafish embryos. [The TCDD-induced *foxq1* gene reported by Planchart & Mattingly [92] was called *foxq1b* in that paper but now has been renamed *foxq1a*. It is located on zebrafish chromosome 2, encodes predicted protein XM_003197808.1, and corresponds to Agilent probe A_15_P199746. The *foxq1* gene identified in the present manuscript was originally called *foxq1*, but now is designated *foxq1b*. It is located on zebrafish chromosome 20, encodes NM_212907.1, and corresponds to Agilent probe A_15_P102120. We will use the currently approved nomenclature of *foxq1a* (the gene reported induced by Planchart & Mattingly) and *foxq1b* (the gene reported to be induced in the current study).]

In the Planchart & Mattingly study [92], *foxq1b* (new nomenclature) was not induced by TCDD (1 nM). However, those authors examined embryos at 24 and 48-hpf. In our studies, *foxq1b* was also not inducible by TCDD (2 nM) at those early times, but became highly inducible at later times (3, 4, and 5 dpf) (**Fig. 4**), demonstrating stage-specific responsiveness not seen with *foxq1a* [92]. The murine *Foxq1* gene also appears to be responsive to TCDD [93], evidence for an evolutionarily conserved role of AHR in regulating *foxq1* genes. Although the zebrafish *foxq1a* gene is expressed in jaw primordia, the site of *foxq1b* expression is not yet known and the functions of these two paralogs in zebrafish development have not yet been investigated. In mammals, FOXQ1 is expressed both in embryos and a variety of adult tissues [94], [95], is required for normal embryonic development [94], [96], and has a recently discovered role in controlling epithelial-mesenchymal transition in human cancer metastasis [97]–[99]. It will be important to better characterize the relationship between *foxq1* and *ahr* genes and their roles in cellular and developmental processes.

### Ontogeny of antioxidant response

Although it is known that the sensitivity of developing vertebrate animals to chemicals varies by developmental stage [60], [100]–[102], the underlying mechanisms are not well understood. Previous research in a variety of vertebrate models has suggested that in early development the antioxidant defense systems are immature and not fully responsive to oxidative stress [33], [103], [104]. However, we are not aware of any systematic investigations of the developmental stages at which vertebrates develop the capacity to respond to oxidative stress by the induction of anti-oxidant defenses. Here, we found that embryos as early as 24 hpf were capable of responding to tBHQ with induction of *gstp1*, *gclc*, and *nrf2a*. The result with *gstp1* differs from that of Kobayashi *et al.* [33], who found by *in situ* hybridization that expression of *gstp1* was inducible at 96 and 120 hpf, but not at 24 hpf. Our results suggest that qRT-PCR is more sensitive for detecting induced *gstp1* at this early time.

Our results also showed that the response to tBHQ varied by developmental time, in a gene-specific manner. For example, *gstp1* and *gclc*, while inducible at 1- and 2-dpf, were less inducible (and more highly variable) at later stages. These results suggest that the set of genes responsive to tBHQ or other oxidants will vary as embryos and later stages develop. This could be related to developmentally programmed changes in GSH redox status [35]. In future studies, it will be important to examine the relationships among GSH status, inducibility of antioxidant defenses, and stage-specific differences in sensitivity to embryotoxicity of oxidant chemicals. Such studies should include an assessment of dose-response relationships and how they may change during development.

### Repression of *mitfa* and pigmentation defects caused by tBHQ

One of the genes that we found by microarray (**Table 4**) and qRT-PCR (**Fig. 4**) to be most strongly repressed in tBHQ-treated embryos and eleutheroembryos was *mitfa*, one of two zebrafish co-orthologs of the mammalian gene *MITF* [61]. Expression of MITF has also been reported to be reduced in mammalian melanocytes exposed to 4-*tert*-butylphenol [105] or melanoma cells exposed to hydrogen peroxide [106]. *MITF* and *mitfa* control melanocyte differentiation and regulate the expression of enzymes involved in melanin synthesis [107]. *mitfa* is defective in zebrafish *nacre* mutants, which exhibit hypopigmentation [62], and knock-down of Mitfa protein in zebrafish embryos with morpholino-modified antisense oligonucleotides causes nearly complete but transient loss of body pigmentation [108], [109]. We found that zebrafish embryos exposed to tBHQ exhibited reduced pigmentation, confirming and extending a previous report [60]. Partial recovery of pigmentation occurred several days after cessation of tBHQ exposure. These results provide a link between oxidative stress, *mitfa*, and loss of pigmentation, and suggest that tBHQ-treated zebrafish embryos could serve as a model for vitiligo, a human skin disease characterized by depigmentation and reduced expression of MITF in melanocytes [110]. Vitiligo has been suggested to have an etiology involving oxidative stress [111] and NRF2 polymorphisms were identified as risk factors in the development of this disease [112].

### hsp70-GFP transgenic zebrafish as tool for screening

Our microarray and qRT-PCR data showed that *hsp70* was induced at all stages and up to 50-fold in zebrafish early life stages exposed to tBHQ (**Table 3** **,** **Fig. 4**). Consistent with this, we found two putative AREs in the promoter of the zebrafish *hsp70* gene, although they were identified only when using a high false discovery rate. The strong response of *hsp70* to tBHQ treatment prompted us to evaluate the effect of tBHQ on embryos of an *hsp70-egfp* transgenic zebrafish line [53], which has been shown previously to respond to heat or cadmium with induced expression of the *egfp* transgene [53], [113]. We found that tBHQ exposure early in development caused induction of EGFP in a restricted pattern, whereas exposure later in development caused widespread EGFP expression (**Fig. 6**).

The widespread induction of *hsp70-egfp* at 4-dpf is consistent with the high level of induction of *hsp70* measured by microarray and qRT-PCR and with studies in human cells showing that *HSP70* is regulated by NRF2 [63]. The strong response of *hsp70*, *hsp40*, *jun*, and *fos* could reflect a direct effect of signaling via ROS, potentiated by GSH depletion [114]. These results demonstrate the potential utility of EGFP transgenic fish for assessing cell- and tissue-specific effects of oxidant chemicals, complementing whole-embryo assessments of gene expression by qRT-PCR and microarray and providing a method for rapidly screening chemicals for the ability to cause oxidative stress during development. However, the *hsp70-egfp* fish respond to a variety of stressors [53], [113], [115], and thus lack the specificity that would be required for a targeted screening assay. Thus, it will be important to develop germ-line transgenic fish lines expressing reporter genes under control of more specific indicators of oxidative stress [43], [65], [66]. The gene expression data reported here will help to identify the appropriate target genes as a source of regulatory elements for use in such an approach.

## Conclusions

The key findings of these experiments are: **1)** embryos are responsive to tBHQ as early as 24-hpf, with strong induction of classical OSR genes like *gstp1* and *gclc*; **2)** the response to tBHQ varies with developmental time, in a gene-specific manner; **3)** at 4-dpf, tBHQ induces a suite of OSR genes including several involved in GSH metabolism, response to DNA damage, amino acid transport, response to hypoxia, iron homeostasis, and inflammation; **4)** microarrays were capable of detecting altered expression of a variety of known and novel oxidant-responsive genes in whole eleutheroembryos; and **5)** patterns of tBHQ-induced gene expression in developing zebrafish exhibit strong similarities but also some differences as compared with genes induced by tBHQ in mammalian systems (adults and cultured cells). These data demonstrate the responsiveness of developing zebrafish to a model oxidant (tBHQ) and illustrate the power of this approach for investigating the mechanisms by which early life stages of vertebrate animals respond to oxidative stress. The results will help guide studies using zebrafish embryonic and larval stages to better understand the chemical and stage specificity of the OSR and its role in determining the sensitivity of vertebrate animals to oxidant chemicals during development.

## Materials and Methods

### Chemicals

2,3,7,8-Tetrachlorodibenzo-*p*-dioxin (TCDD) was obtained from Ultra Scientific (Hope, RI). Dimethyl sulfoxide (DMSO) was from Sigma-Aldrich (St. Louis, MO). *Tert*-butylhydroquinone (tBHQ) was obtained from Acros Organics (Geel, Belgium).

### Embryo culture

For experiments 1 and 2, we used adult zebrafish of the TL strain, a generous gift of Dr. Mark Fishman (Massachusetts General Hospital, Cambridge, MA), to generate embryos. Fish were maintained as described previously [116]. For experiment 3, we used hsp70-EGFP fish [*Tg(hsp70l:EGFP)_unspecified*] [53], a generous gift from Dr. John Y. Kuwada (University of Michigan).

The experiments were carried out according to the recommendations in the *Guide for the Care and Use of Laboratory Animals* of the National Institutes of Health. The protocols were approved by the Institutional Animal Care and Use Committee of the Woods Hole Oceanographic Institution (IACUC Assurance: A3630-01).

### Exposure of embryos, eleutheroembryos, or larvae to chemicals

#### Experiment 1

Separate groups of 30 embryos generated from TL adults were placed in 20 ml system water in 10-cm glass petri dishes. At 1, 2, 3, 4, 5, and 6-dpf, embryos, eleutheroembryos, or larvae were exposed for 6 hr to DMSO (0.1%), TCDD (2 nM), or tBHQ (10 µM) (4 groups per compound per time point) (**Fig. 1**). The concentration of TCDD (2 nM) is known to produce strong induction of gene expression in developing zebrafish [51], [60]. The concentration of tBHQ (10 µM) is one at which strong induction of *gstp1* was reported in 4-dpf eleutheroembryos after 6 hr exposure [33]; higher concentrations (30 µM) caused acute toxicity in our hands. Embryos were frozen immediately after the 6-hr exposure. The short exposure time was chosen to select for primary (direct) responses and minimize changes in gene expression that are secondary to toxic effects. RNA was isolated for real-time, quantitative reverse-transcription-polymerase chain reaction (qRT-PCR) and microarray analysis, as described below. (Throughout the paper, we refer to these embryos by the age when the 6-hr exposure was initiated, e.g. 4-dpf eleutheroembryos refers to eleutheroembryos that were exposed to tBHQ for 6 hr beginning at 4-dpf.)

#### Experiment 2

Groups of 150 TL embryos were exposed to tBHQ (5 or 10 µM) or 0.1% DMSO in glass petri dishes containing 20 ml of 0.3× Danieau's from 32–47 hpf, then washed in 0.3× Danieau's. At 52 hpf, randomly selected embryos were mounted in a left-lateral orientation in 3% methylcellulose and imaged using on a Zeiss dissecting scope with a Zeiss Axiocam MR color CCD camera. Embryos were subsequently maintained in 0.3× Danieau's water at 28.5 C until 120 hpf, when they were imaged again in order to assess recovery of pigmentation.

#### Experiment 3

Separate groups of 1-dpf embryos (n = 30) from hsp70-EGFP transgenic fish were exposed to DMSO (0.1%) or tBHQ (10 µM) for 4 hrs at 28°C. After exposure, embryos were washed and held for 4 additional hours in 0.3× Danieau's before being inspected for EGFP expression by fluorescence microscopy using an Axiovert 200 inverted microscope with Zeiss Filter Set 38 HE (489038; excitation BP 470/40, FT 495, emission BP 525/50). Another experiment was performed by exposing 4-dpf eleutheroembryos (n = 15) to DMSO (0.1%) or tBHQ (10 µM) for 4 hrs at 28°C. After exposure, eleutheroembryos were washed with 0.3× Danieau's and inspected by fluorescence microscopy at 4 hrs post-exposure (104 hpf) to assess EGFP expression.

### RNA isolation and qRT-PCR

Total RNA was isolated using RNA STAT-60 (Tel-Test B, Inc., Friendswood, TX) and DNase-treated using the Turbo DNA-*free* kit (Ambion, Austin, TX). Poly(A)+ RNA was purified using the MicroPoly(A)Purist Kit (Ambion). cDNA was synthesized from 2 µg of total RNA using Omniscript reverse transcriptase (Qiagen, Valencia, CA). Real-time qRT-PCR was performed using the iQ SYBR Green Supermix (Bio-Rad, Hercules, CA) in an iCycler iQ Real-Time PCR Detection System (Bio-Rad) as described previously [117]. Primers were synthesized by Midland Certified Reagent Company, Midland, TX. Primer sequences can be found in **Table S1**.

### Microarray analyses

We examined a subset of RNA samples from Experiment 1 by microarray using the Agilent 22k long-oligo zebrafish array. We analyzed all of the 4-dpf samples: four biological replicates each for DMSO-, tBHQ- and TCDD-treated eleutheroembryos, each hybridized against a universal reference mRNA created from equal amounts of RNA from 2 replicates each from all toxicants (TCDD, tBHQ, DMSO) and time points (1, 2, 3, 4, 5, 6-dpf). The use of a universal reference RNA balances efficiency with statistical power [118] and has several advantages [119]–[121]. It facilitates normalization because all of the genes expressed in experimental samples are represented in the reference samples [122]. Dye bias is minimized because all experimental samples are labeled with the same dye; thus, dye swaps are not needed [121]. To verify this, we performed quality control hybridizations including a dye swap and a self-self hybridization. Analysis of a self-self hybridization of the Universal Reference (composed of equal amounts of RNA from all timepoints, toxicants, and replicates) revealed 18293 of 21495 features (85%) with signal above background (calculated as 2.6 times the background standard deviation). This indicates that the majority of the probes on the Agilent microarray represent transcripts expressed in 1–6 dpf embryos.

RNA samples from 4-dpf eleutheroembryos treated with DMSO, tBHQ, or TCDD were checked for quality using a NanoDrop ND-1000 spectrophotometer and an Agilent 2100 BioAnalyzer. cDNA synthesis from 200 ng of total RNA was performed using the Agilent Low RNA Input Linear Fluorescent Amplification Plus kit, following the manufacturer's instructions. cRNA was synthesized from the cDNA template, with incorporation of either cyanine-5-CTP or cyanine-3-CTP (Perkin Elmer). Labeled cRNA was purified using the Qiagen RNeasy Mini kit; quantity and quality was assessed by NanoDrop spectrophotometer and Agilent BioAnalyzer. cRNA samples were hybridized to Agilent 22k zebrafish microarrays using the Agilent Gene Expression Hybridization Kit. An aliquot (750 ng) of each cy5-labeled sample cRNA was hybridized against 750 ng of cy3-labeled cRNA derived from the Universal mRNA Reference. Labeled cRNAs were combined with the Agilent 25× fragmentation buffer and incubated at 60°C for 30 minutes. This was followed by mixing with 2× hybridization buffer, after which 100 µl of the product was spread evenly across the surface of an Agilent 22K zebrafish microarray. The loaded microarray was incubated at 60°C for 17 hours with rotation in an Agilent DNA Microarray Hybridization Oven. Post-hybridization, microarray slides were washed, air-dried, and stored in darkness with desiccation prior to laser-excited fluorescence scanning in an Agilent DNA Microarray Scanner.

Analysis of raw microarray data was performed using Agilent's feature extraction protocol, which includes spot finding, spot analysis, background subtraction (using local background plus global background based on spots along the central tendency line for red versus green intensity), dye normalization (linear and lowess algorithms, using spots along the central tendency line as for background subtraction), and final calculation of Cy5/Cy3 ratios and log_2_ transformed fold change for each spot. Features with signal not significantly above background, non-uniform features, and features exhibiting saturation were flagged. The microarray data have been deposited in MIAME-compliant format in the Gene Expression Omnibus (GEO) database at the U.S. National Center for Biotechnology Information (accession number GSE10157; http://www.ncbi.nlm.nih.gov/geo/).

Probes indicating significantly different relative transcript abundance among the DMSO, TCDD, and tBHQ treatments were determined by Analysis of Variance (ANOVA) using MEV in the TM4 suite of microarray software [123], [124]. Data were first log transformed and values for each probe median centered. ANOVA was performed with a distribution based on 1000 permutations of the data, a significance value of p<0.05, and control of False Discovery Rate (FDR) at 5% [125], [126]. An objective of the statistical analysis was to minimize type II error while maintaining a reasonable false discovery rate.

Hierarchical cluster analysis of the significant probes was performed using CLUSTER software [127] and log_2_ transformed fold change values were median centered for both probes and microarrays. Cluster analysis used Pearson's correlation (i.e. centered) and average linkage clustering. Enrichment of Gene Ontology (GO) terms for clusters of significant probes thus identified was examined using the FatiGO+ software [56]. FatiGO+ uses GO terms assigned within the Ensembl annotation of the zebrafish genome [128] and the background set of probes used in each analysis was the entire probe set of the Agilent microarray less the probes found significant by the ANOVA analysis described above.

### Annotation of probes on the array

To aid interpretation of all results, we updated the microarray annotation provided by Agilent by incorporating annotations, functional domain predictions, and Gene Ontology assignments available at Ensembl (version 48, based on assembly Zv7), ZFIN, UniProt, RefSeq, and the Harvard Gene Index Annotation. Additional putative annotations were obtained from Ensembl's Integr8 project [129], which attempts annotation of proteins based upon putative orthology among organisms. Comparison to the latest Zv9 assembly assignment of Agilent probes to genes at Ensembl did not improve gene annotations.

A number of probes found important in our statistical analyses were manually annotated by searching the zebrafish RefSeq RNA database or available zebrafish ESTs for the putative transcript and examining the similarity of the encoded protein to those of other model organisms by using BLAST against the GenBank non-redundant protein database.

### Prediction of *cis*-regulatory elements

The 10 kb upstream regions of all genes predicted by Ensembl (version 59, genome assembly Zv9) were searched for putative *cis*-regulatory motifs using the FIMO software [130]. Searches were for motif matches in either orientation/strand in the target upstream region and were based on position-specific scoring matrix (PSSM) searches with p<0.0001 and associated calculation of q-values [131], with correction for background nucleotide frequencies of complete nuclear genome sequences. Background frequencies were estimated by examining the complete nuclear chromosome data of the Zv9 assembly. Searches used PSSM models for ARE [132], DRE [133], ERE [134], NRF2 [135], PXR [136] and a subset of the JASPAR database [137]: Arnt, Arnt::Ahr, NF-kappaB, SP1, NFE2L1::MafG, AP1, REL, NFKB1, RELA, Mafb, NFE2L2, HIF1A::ARNT. We focused our attention on significant hits (p<0.0001) with false-discovery rate of 10% or less.

## Supporting Information

Table S1 **Primers for Real-time RT-PCR.**
(DOCX)

Table S2 **List of all significant probes and the ratios (tBHQ/DMSO, TCDD/DMSO).**
(XLS)

Table S3 **Enrichment of Gene Ontology (GO) terms for Ensembl annotated probes with significantly different abundance between TCDD, tBHQ, and DMSO treated eleutheroembryos.**
(DOC)

Table S4 **List of 2-fold significant probes.**
(XLS)

Table S5 **Comparison of altered gene expression as measured by microarray and qRT-PCR.**
(DOC)

## References

[1] Hansen JM, Go YM, Jones DP (2006) Nuclear and mitochondrial compartmentation of oxidative stress and redox signaling. Annu Rev Pharmacol Toxicol 46: 215–234. 16402904 10.1146/annurev.pharmtox.46.120604.141122

[2] Cooke MS, Evans MD, Dizdaroglu M, Lunec J (2003) Oxidative DNA damage: mechanisms, mutation, and disease. Faseb J 17: 1195–1214. 12832285 10.1096/fj.02-0752rev

[3] National Research Council/National Academy of Sciences (2000) Scientific frontiers in developmental toxicology and risk assessment: National Academy Press, Washington, DC, 312 pages. 25077274

[4] Rangasamy T, Cho CY, Thimmulappa RK, Zhen L, Srisuma SS, et al (2004) Genetic ablation of Nrf2 enhances susceptibility to cigarette smoke-induced emphysema in mice. J Clin Invest 114: 1248–1259. 15520857 10.1172/JCI21146 PMC524225

[5] Andersen JK (2004) Oxidative stress in neurodegeneration: cause or consequence? Nat Med 10 Suppl: S18–25. 15298006 10.1038/nrn1434

[6] Bossy-Wetzel E, Schwarzenbacher R, Lipton SA (2004) Molecular pathways to neurodegeneration. Nat Med 10 Suppl: S2–9. 15272266 10.1038/nm1067

[7] Dennery PA (2004) Role of redox in fetal development and neonatal diseases. Antioxid Redox Signal 6: 147–153. 14713346 10.1089/152308604771978453

[8] Hernandez-Garcia D, Wood CD, Castro-Obregon S, Covarrubias L (2010) Reactive oxygen species: A radical role in development? Free Radic Biol Med 49: 130–143. 20353819 10.1016/j.freeradbiomed.2010.03.020

[9] Wells PG, Bhuller Y, Chen CS, Jeng W, Kasapinovic S, et al (2005) Molecular and biochemical mechanisms in teratogenesis involving reactive oxygen species. Toxicol Appl Pharmacol 207: 354–366. 16081118 10.1016/j.taap.2005.01.061

[10] Perera FP, Tang D, Tu YH, Cruz LA, Borjas M, et al (2004) Biomarkers in maternal and newborn blood indicate heightened fetal susceptibility to procarcinogenic DNA damage. Environ Health Perspect 112: 1133–1136. 15238289 10.1289/ehp.6833 PMC1247389

[11] Wells PG, Kim PM, Laposa RR, Nicol CJ, Parman T, et al (1997) Oxidative damage in chemical teratogenesis. Mutat Res 396: 65–78. 9434860 10.1016/s0027-5107(97)00175-9

[12] Wells PG, Winn LM (1996) Biochemical toxicology of chemical teratogenesis. Crit Rev Biochem Mol Biol 31: 1–40. 8744954 10.3109/10409239609110574

[13] Reimers MJ, La Du JK, Periera CB, Giovanini J, Tanguay RL (2006) Ethanol-dependent toxicity in zebrafish is partially attenuated by antioxidants. Neurotoxicol Teratol 28: 497–508. 16904866 10.1016/j.ntt.2006.05.007

[14] Zhu X, Zhu L, Li Y, Duan Z, Chen W, et al (2007) Developmental toxicity in zebrafish (Danio rerio) embryos after exposure to manufactured nanomaterials: buckminsterfullerene aggregates (nC60) and fullerol. Environ Toxicol Chem 26: 976–979. 17521145 10.1897/06-583.1

[15] Loeken MR (2006) Advances in understanding the molecular causes of diabetes-induced birth defects. J Soc Gynecol Investig 13: 2–10. 10.1016/j.jsgi.2005.09.007 16303321

[16] Ornoy A (2007) Embryonic oxidative stress as a mechanism of teratogenesis with special emphasis on diabetic embryopathy. Reprod Toxicol 24: 31–41. 17548185 10.1016/j.reprotox.2007.04.004

[17] Osburn WO, Kensler TW (2008) Nrf2 signaling: an adaptive response pathway for protection against environmental toxic insults. Mutat Res 659: 31–39. 18164232 10.1016/j.mrrev.2007.11.006 PMC2585047

[18] Kensler TW, Wakabayashi N, Biswal S (2007) Cell Survival Responses to Environmental Stresses Via the Keap1-Nrf2-ARE Pathway. Annu Rev Pharmacol Toxicol 47: 89–116. 16968214 10.1146/annurev.pharmtox.46.120604.141046

[19] Wells PG, McCallum GP, Chen CS, Henderson JT, Lee CJ, et al (2009) Oxidative stress in developmental origins of disease: teratogenesis, neurodevelopmental deficits, and cancer. Toxicol Sci 108: 4–18. 19126598 10.1093/toxsci/kfn263

[20] Hansen JM (2006) Oxidative stress as a mechanism of teratogenesis. Birth Defects Res C Embryo Today 78: 293–307. 17315243 10.1002/bdrc.20085

[21] Timme-Laragy AR, Karchner SI, Franks DG, Jenny MJ, Harbeitner RC, et al (2012) Nrf2b: a novel zebrafish paralog of the oxidant-responsive transcription factor NF-E2-related factor 2 (NRF2). J Biol Chem 287: 4609–4627. 22174413 10.1074/jbc.M111.260125 PMC3281635

[22] Dinkova-Kostova AT, Holtzclaw WD, Kensler TW (2005) The role of Keap1 in cellular protective responses. Chem Res Toxicol 18: 1779–1791. 16359168 10.1021/tx050217c

[23] Nguyen T, Sherratt PJ, Pickett CB (2003) Regulatory mechanisms controlling gene expression mediated by the antioxidant response element. Annu Rev Pharmacol Toxicol 43: 233–260. 12359864 10.1146/annurev.pharmtox.43.100901.140229

[24] Nguyen T, Nioi P, Pickett CB (2009) The Nrf2-antioxidant response element signaling pathway and its activation by oxidative stress. J Biol Chem 284: 13291–13295. 19182219 10.1074/jbc.R900010200 PMC2679427

[25] Teraoka H, Dong W, Hiraga T (2003) Zebrafish as a novel experimental model for developmental toxicology. Congenit Anom Kyoto 43: 123–132. 12893971 10.1111/j.1741-4520.2003.tb01036.x

[26] Spitsbergen JM, Kent ML (2003) The state of the art of the zebrafish model for toxicology and toxicologic pathology research–advantages and current limitations. Toxicol Pathol 31 Suppl: 62–87. 12597434 10.1080/01926230390174959 PMC1909756

[27] Hill AJ, Teraoka H, Heideman W, Peterson RE (2005) Zebrafish as a model vertebrate for investigating chemical toxicity. Toxicol Sci 86: 6–19. 15703261 10.1093/toxsci/kfi110

[28] Embry MR, Belanger SE, Braunbeck TA, Galay-Burgos M, Halder M, et al (2010) The fish embryo toxicity test as an animal alternative method in hazard and risk assessment and scientific research. Aquat Toxicol 97: 79–87. 20061034 10.1016/j.aquatox.2009.12.008

[29] Selderslaghs IW, Van Rompay AR, De Coen W, Witters HE (2009) Development of a screening assay to identify teratogenic and embryotoxic chemicals using the zebrafish embryo. Reprod Toxicol 28: 308–320. 19447169 10.1016/j.reprotox.2009.05.004

[30] Strahle U, Scholz S, Geisler R, Greiner P, Hollert H, et al (2012) Zebrafish embryos as an alternative to animal experiments–a commentary on the definition of the onset of protected life stages in animal welfare regulations. Reprod Toxicol 33: 128–132. 21726626 10.1016/j.reprotox.2011.06.121

[31] Kelly KA, Havrilla CM, Brady TC, Abramo KH, Levin ED (1998) Oxidative stress in toxicology: established mammalian and emerging piscine model systems. Environ Health Perspect 106: 375–384. 9637794 10.1289/ehp.98106375 PMC1533135

[32] Carvan MJ III, Solis WA, Gedamu L, Nebert DW (2000) Activation of transcription factors in zebrafish cell cultures by environmental pollutants. Arch Biochem Biophys 376: 320–327. 10775418 10.1006/abbi.2000.1727

[33] Kobayashi M, Itoh K, Suzuki T, Osanai H, Nishikawa K, et al (2002) Identification of the interactive interface and phylogenic conservation of the Nrf2-Keap1 system. Genes Cells 7: 807–820. 12167159 10.1046/j.1365-2443.2002.00561.x

[34] Di Giulio RT, Meyer JN (2007) Chapter 6. Reactive Oxygen Species and Oxidative Stress. In: Di Giulio RT, Hinton DE, editors. The Toxicology of Fishes: Taylor & Francis. pp. 273–324.

[35] Timme-Laragy AR, Goldstone JV, Imhoff BR, Stegeman JJ, Hahn ME, et al (2013) Glutathione redox dynamics and expression of glutathione-related genes in the developing embryo. Free Radic Biol Med 65C: 89–101. 10.1016/j.freeradbiomed.2013.06.011 PMC3823629 23770340

[36] Fang L, Miller YI (2012) Emerging applications for zebrafish as a model organism to study oxidative mechanisms and their roles in inflammation and vascular accumulation of oxidized lipids. Free Radic Biol Med 53: 1411–1420. 22906686 10.1016/j.freeradbiomed.2012.08.004 PMC3448821

[37] Wang L, Gallagher EP (2013) Role of Nrf2 antioxidant defense in mitigating cadmium-induced oxidative stress in the olfactory system of zebrafish. Toxicol Appl Pharmacol 266: 177–186. 23174481 10.1016/j.taap.2012.11.010

[38] Timme-Laragy AR, Van Tiem LA, Linney EA, Di Giulio RT (2009) Antioxidant responses and NRF2 in synergistic developmental toxicity of PAHs in zebrafish. Toxicol Sci 109: 217–227. 19233942 10.1093/toxsci/kfp038 PMC2721659

[39] Kobayashi M, Li L, Iwamoto N, Nakajima-Takagi Y, Kaneko H, et al (2009) The antioxidant defense system Keap1-Nrf2 comprises a multiple sensing mechanism for responding to a wide range of chemical compounds. Mol Cell Biol 29: 493–502. 19001094 10.1128/MCB.01080-08 PMC2612520

[40] Li L, Kobayashi M, Kaneko H, Nakajima-Takagi Y, Nakayama Y, et al (2008) Molecular Evolution of Keap1: Two Keap1 molecules with distinctive intervening region structures are conserved among fish. J Biol Chem 283: 3248–3255. 18057000 10.1074/jbc.M708702200

[41] Carvan MJ III, Sonntag DM, Cmar CB, Cook RS, Curran MA, et al (2001) Oxidative stress in zebrafish cells: potential utility of transgenic zebrafish as a deployable sentinel for site hazard ranking. Science of the Total Environment 274: 183–196. 11453295 10.1016/s0048-9697(01)00742-2

[42] Suzuki T, Takagi Y, Osanai H, Li L, Takeuchi M, et al (2005) Pi class glutathione S-transferase genes are regulated by Nrf 2 through an evolutionarily conserved regulatory element in zebrafish. Biochem J 388: 65–73. 15654768 10.1042/BJ20041860 PMC1186694

[43] Kusik BW, Carvan MJ 3rd, Udvadia AJ (2008) Detection of mercury in aquatic environments using EPRE reporter zebrafish. Mar Biotechnol (NY) 10: 750–757. 18537037 10.1007/s10126-008-9113-x PMC8898586

[44] Meyer A, Van de Peer Y (2005) From 2R to 3R: evidence for a fish-specific genome duplication (FSGD). Bioessays 27: 937–945. 16108068 10.1002/bies.20293

[45] Postlethwait J, Amores A, Cresko W, Singer A, Yan YL (2004) Subfunction partitioning, the teleost radiation and the annotation of the human genome. Trends Genet 20: 481–490. 15363902 10.1016/j.tig.2004.08.001

[46] Williams LM, Timme-Laragy AR, Goldstone JV, McArthur AG, Stegeman JJ, et al (2013) Developmental expression of the Nfe2-related factor (Nrf) transcription factor family. PLoS ONE 8: e79574. 24298298 10.1371/journal.pone.0079574 PMC3840143

[47] Li J, Spletter ML, Johnson JA (2005) Dissecting tBHQ induced ARE-driven gene expression through long and short oligonucleotide arrays. Physiol Genomics 21: 43–58. 15613614 10.1152/physiolgenomics.00214.2004

[48] Han ES, Muller FL, Perez VI, Qi W, Liang H, et al (2008) The in vivo gene expression signature of oxidative stress. Physiol Genomics 34: 112–126. 18445702 10.1152/physiolgenomics.00239.2007 PMC2532791

[49] Belanger SE, Balon EK, Rawlings JM (2010) Saltatory ontogeny of fishes and sensitive early life stages for ecotoxicology tests. Aquat Toxicol 97: 88–95. 20042243 10.1016/j.aquatox.2009.11.020

[50] Carney SA, Chen J, Burns CG, Xiong KM, Peterson RE, et al (2006) Aryl hydrocarbon receptor activation produces heart-specific transcriptional and toxic responses in developing zebrafish. Mol Pharmacol 70: 549–561. 16714409 10.1124/mol.106.025304

[51] Handley-Goldstone HM, Grow MW, Stegeman JJ (2005) Cardiovascular gene expression profiles of dioxin exposure in zebrafish embryos. Toxicol Sci 85: 683–693. 15716485 10.1093/toxsci/kfi116

[52] Goldstone HM, Stegeman JJ (2006) Molecular mechanisms of 2,3,7,8-tetrachlorodibenzo-p-dioxin cardiovascular embryotoxicity. Drug Metab Rev 38: 261–289. 16684661 10.1080/03602530600570099

[53] Halloran MC, Sato-Maeda M, Warren JT, Su F, Lele Z, et al (2000) Laser-induced gene expression in specific cells of transgenic zebrafish. Development 127: 1953–1960. 10751183 10.1242/dev.127.9.1953

[54] Miao W, Hu L, Scrivens PJ, Batist G (2005) Transcriptional regulation of NF-E2 p45-related factor (NRF2) expression by the aryl hydrocarbon receptor-xenobiotic response element signaling pathway: direct cross-talk between phase I and II drug-metabolizing enzymes. J Biol Chem 280: 20340–20348. 15790560 10.1074/jbc.M412081200

[55] Fletcher N, Wahlstrom D, Lundberg R, Nilsson CB, Nilsson KC, et al (2005) 2,3,7,8-Tetrachlorodibenzo-p-dioxin (TCDD) alters the mRNA expression of critical genes associated with cholesterol metabolism, bile acid biosynthesis, and bile transport in rat liver: a microarray study. Toxicol Appl Pharmacol 207: 1–24. 10.1016/j.taap.2004.12.003 16054898

[56] Al-Shahrour F, Minguez P, Tarraga J, Medina I, Alloza E, et al (2007) FatiGO+: a functional profiling tool for genomic data. Integration of functional annotation, regulatory motifs and interaction data with microarray experiments. Nucleic Acids Res 35: W91–96. 17478504 10.1093/nar/gkm260 PMC1933151

[57] Yan YL, Willoughby J, Liu D, Crump JG, Wilson C, et al (2005) A pair of Sox: distinct and overlapping functions of zebrafish sox9 co-orthologs in craniofacial and pectoral fin development. Development 132: 1069–1083. 15689370 10.1242/dev.01674

[58] Yokoi H, Yan YL, Miller MR, BreMiller RA, Catchen JM, et al (2009) Expression profiling of zebrafish sox9 mutants reveals that Sox9 is required for retinal differentiation. Dev Biol 329: 1–15. 19210963 10.1016/j.ydbio.2009.01.002 PMC2706370

[59] Arikawa E, Sun Y, Wang J, Zhou Q, Ning B, et al (2008) Cross-platform comparison of SYBR Green real-time PCR with TaqMan PCR, microarrays and other gene expression measurement technologies evaluated in the MicroArray Quality Control (MAQC) study. BMC Genomics 9: 328. 18620571 10.1186/1471-2164-9-328 PMC2491643

[60] Yang L, Kemadjou JR, Zinsmeister C, Legradi J, Bauer M, et al (2007) Transcriptional profiling reveals barcode-like toxicogenomic responses in the zebrafish embryo. Genome Biol 8: R227. 17961207 10.1186/gb-2007-8-10-r227 PMC2246301

[61] Lister JA, Close J, Raible DW (2001) Duplicate mitf genes in zebrafish: complementary expression and conservation of melanogenic potential. Dev Biol 237: 333–344. 11543618 10.1006/dbio.2001.0379

[62] Lister JA, Robertson CP, Lepage T, Johnson SL, Raible DW (1999) nacre encodes a zebrafish microphthalmia-related protein that regulates neural-crest-derived pigment cell fate. Development 126: 3757–3767. 10433906 10.1242/dev.126.17.3757

[63] Zhang X, Lu L, Dixon C, Wilmer W, Song H, et al (2004) Stress protein activation by the cyclopentenone prostaglandin 15-deoxy-delta12,14-prostaglandin J2 in human mesangial cells. Kidney Int 65: 798–810. 14871400 10.1111/j.1523-1755.2004.00454.x

[64] Blechinger SR, Evans TG, Tang PT, Kuwada JY, Warren JT Jr, et al (2002) The heat-inducible zebrafish hsp70 gene is expressed during normal lens development under non-stress conditions. Mech Dev 112: 213–215. 11850198 10.1016/s0925-4773(01)00652-9

[65] Tsujita T, Li L, Nakajima H, Iwamoto N, Nakajima-Takagi Y, et al (2011) Nitro-fatty acids and cyclopentenone prostaglandins share strategies to activate the Keap1-Nrf2 system: a study using green fluorescent protein transgenic zebrafish. Genes Cells 16: 46–57. 21143560 10.1111/j.1365-2443.2010.01466.x PMC4124525

[66] Nakajima H, Nakajima-Takagi Y, Tsujita T, Akiyama S, Wakasa T, et al (2011) Tissue-restricted expression of Nrf2 and its target genes in zebrafish with gene-specific variations in the induction profiles. PLoS ONE 6: e26884. 22046393 10.1371/journal.pone.0026884 PMC3201981

[67] Dalton TP, Chen Y, Schneider SN, Nebert DW, Shertzer HG (2004) Genetically altered mice to evaluate glutathione homeostasis in health and disease. Free Radic Biol Med 37: 1511–1526. 15477003 10.1016/j.freeradbiomed.2004.06.040

[68] Rojas E, Valverde M, Kala SV, Kala G, Lieberman MW (2000) Accumulation of DNA damage in the organs of mice deficient in gamma-glutamyltranspeptidase. Mutat Res 447: 305–316. 10751614 10.1016/s0027-5107(99)00191-8

[69] Usenko CY, Harper SL, Tanguay RL (2008) Fullerene C60 exposure elicits an oxidative stress response in embryonic zebrafish. Toxicol Appl Pharmacol 229: 44–55. 18299140 10.1016/j.taap.2007.12.030 PMC2421009

[70] Gharavi N, El-Kadi AO (2005) tert-Butylhydroquinone is a novel aryl hydrocarbon receptor ligand. Drug Metab Dispos 33: 365–372. 15608132 10.1124/dmd.104.002253

[71] Schreiber TD, Kohle C, Buckler F, Schmohl S, Braeuning A, et al (2006) Regulation of CYP1A1 gene expression by the antioxidant tert-butylhydroquinone. Drug Metab Dispos 34: 1096–1101. 16581943 10.1124/dmd.106.009662

[72] Gracey AY, Troll JV, Somero GN (2001) Hypoxia-induced gene expression profiling in the euryoxic fish Gillichthys mirabilis. Proc Natl Acad Sci U S A 98: 1993–1998. 11172064 10.1073/pnas.98.4.1993 PMC29370

[73] Zhang QH, Ye M, Wu XY, Ren SX, Zhao M, et al (2000) Cloning and functional analysis of cDNAs with open reading frames for 300 previously undefined genes expressed in CD34+ hematopoietic stem/progenitor cells. Genome Res 10: 1546–1560. 11042152 10.1101/gr.140200 PMC310934

[74] Fan F, Jin S, Amundson SA, Tong T, Fan W, et al (2002) ATF3 induction following DNA damage is regulated by distinct signaling pathways and over-expression of ATF3 protein suppresses cells growth. Oncogene 21: 7488–7496. 12386811 10.1038/sj.onc.1205896

[75] Fiermonte G, De Leonardis F, Todisco S, Palmieri L, Lasorsa FM, et al (2004) Identification of the mitochondrial ATP-Mg/Pi transporter. Bacterial expression, reconstitution, functional characterization, and tissue distribution. J Biol Chem 279: 30722–30730. 15123600 10.1074/jbc.M400445200

[76] Nakamichi N, Kambe Y, Oikawa H, Ogura M, Takano K, et al (2005) Protection by exogenous pyruvate through a mechanism related to monocarboxylate transporters against cell death induced by hydrogen peroxide in cultured rat cortical neurons. J Neurochem 93: 84–93. 15773908 10.1111/j.1471-4159.2005.02999.x

[77] Takabe W, Kanai Y, Chairoungdua A, Shibata N, Toi S, et al (2004) Lysophosphatidylcholine enhances cytokine production of endothelial cells via induction of L-type amino acid transporter 1 and cell surface antigen 4F2. Arterioscler Thromb Vasc Biol 24: 1640–1645. 15178563 10.1161/01.ATV.0000134377.17680.26

[78] Lee JI, Dominy JE Jr, Sikalidis AK, Hirschberger LL, Wang W, et al (2008) HepG2/C3A cells respond to cysteine deprivation by induction of the amino acid deprivation/integrated stress response pathway. Physiol Genomics 33: 218–229. 18285520 10.1152/physiolgenomics.00263.2007

[79] Escartin C, Joon Won S, Malgorn C, Auregan G, Berman AE, et al (2011) Nuclear factor erythroid 2-related factor 2 facilitates neuronal glutathione synthesis by upregulating neuronal excitatory amino Acid transporter 3 expression. J Neurosci 31: 7392–7401. 21593323 10.1523/JNEUROSCI.6577-10.2011 PMC3339848

[80] Harding HP, Zhang Y, Zeng H, Novoa I, Lu PD, et al (2003) An integrated stress response regulates amino acid metabolism and resistance to oxidative stress. Mol Cell 11: 619–633. 12667446 10.1016/s1097-2765(03)00105-9

[81] Konwinski RR, Haddad R, Chun JA, Klenow S, Larson SC, et al (2004) Oltipraz, 3H-1,2-dithiole-3-thione, and sulforaphane induce overlapping and protective antioxidant responses in murine microglial cells. Toxicol Lett 153: 343–355. 15454310 10.1016/j.toxlet.2004.06.006

[82] Agyeman AS, Chaerkady R, Shaw PG, Davidson NE, Visvanathan K, et al (2011) Transcriptomic and proteomic profiling of KEAP1 disrupted and sulforaphane-treated human breast epithelial cells reveals common expression profiles. Breast Cancer Res Treat 132: 175–187.. 21597922 10.1007/s10549-011-1536-9 PMC3564494

[83] Yates MS, Tran QT, Dolan PM, Osburn WO, Shin S, et al (2009) Genetic versus chemoprotective activation of Nrf2 signaling: overlapping yet distinct gene expression profiles between Keap1 knockout and triterpenoid-treated mice. Carcinogenesis 30: 1024–1031. 19386581 10.1093/carcin/bgp100 PMC2691141

[84] Reisman SA, Yeager RL, Yamamoto M, Klaassen CD (2009) Increased Nrf2 activation in livers from Keap1-knockdown mice increases expression of cytoprotective genes that detoxify electrophiles more than those that detoxify reactive oxygen species. Toxicol Sci 108: 35–47. 19129213 10.1093/toxsci/kfn267 PMC2644398

[85] Hahn ME, Karchner SI, Franks DG, Timme-Laragy AR, McArthur AG (2014) Chemical-Specific Oxidative Stress Response in Zebrafish Embryos. Toxicol Sci (The Toxicologist Supplement) 52 (Abstract #209)

[86] Reichard JF, Dalton TP, Shertzer HG, Puga A (2005) Induction of Oxidative Stress Responses by Dioxin and other Ligands of the Aryl Hydrocarbon Receptor. Dose Response 3: 306–331. 10.2203/dose-response.003.03.003 PMC2475945 18648615

[87] Boverhof DR, Burgoon LD, Tashiro C, Chittim B, Harkema JR, et al (2005) Temporal and dose-dependent hepatic gene expression patterns in mice provide new insights into TCDD-Mediated hepatotoxicity. Toxicol Sci 85: 1048–1063. 15800033 10.1093/toxsci/kfi162

[88] Dalton T, Puga A, Shertzer H (2002) Induction of cellular oxidative stress by aryl hydrocarbon receptor activation. Chem Biol Interact 141: 77. 12213386 10.1016/s0009-2797(02)00067-4

[89] Alexeyenko A, Wassenberg DM, Lobenhofer EK, Yen J, Sonnhammer EL, et al (2010) Dynamic Zebrafish Interactome Reveals Transcriptional Mechanisms of Dioxin Toxicity. PLoS ONE 5: e10465. 20463971 10.1371/journal.pone.0010465 PMC2864754

[90] Hahn ME, Karchner SI, Franks DG, Woodin BR, Barott KL, et al (2007) The transcriptional response to oxidative stress in zebrafish embryos. Toxicol Sci (The Toxicologist Supplement) 96: 326–327 (Abstract #1578)

[91] Hahn ME, Karchner SI, Franks DG, Woodin BR, Barott KL, et al (2008) The transcriptional response to oxidative stress in fish embryos and cells exposed to tert-butylhydroquinone (tBHQ) or 2,3,7,8-tetrachlorodibenzo-p-dioxin (TCDD). Mar Environ Res 66: 138.

[92] Planchart A, Mattingly CJ (2010) 2,3,7,8-Tetrachlorodibenzo-p-dioxin upregulates FoxQ1b in zebrafish jaw primordium. Chem Res Toxicol 23: 480–487. 20055451 10.1021/tx9003165 PMC2839046

[93] Frericks M, Burgoon LD, Zacharewski TR, Esser C (2008) Promoter analysis of TCDD-inducible genes in a thymic epithelial cell line indicates the potential for cell-specific transcription factor crosstalk in the AhR response. Toxicol Appl Pharmacol 232: 268–279. 18691609 10.1016/j.taap.2008.07.009

[94] Hong HK, Noveroske JK, Headon DJ, Liu T, Sy MS, et al (2001) The winged helix/forkhead transcription factor Foxq1 regulates differentiation of hair in satin mice. Genesis 29: 163–171. 11309849 10.1002/gene.1020

[95] Bieller A, Pasche B, Frank S, Glaser B, Kunz J, et al (2001) Isolation and characterization of the human forkhead gene FOXQ1. DNA Cell Biol 20: 555–561. 11747606 10.1089/104454901317094963

[96] Goering W, Adham IM, Pasche B, Manner J, Ochs M, et al (2008) Impairment of gastric acid secretion and increase of embryonic lethality in Foxq1-deficient mice. Cytogenet Genome Res 121: 88–95. 18544931 10.1159/000125833

[97] Zhang H, Meng F, Liu G, Zhang B, Zhu J, et al (2011) Forkhead transcription factor foxq1 promotes epithelial-mesenchymal transition and breast cancer metastasis. Cancer Res 71: 1292–1301. 21285253 10.1158/0008-5472.CAN-10-2825 PMC3906209

[98] Qiao Y, Jiang X, Lee ST, Karuturi RK, Hooi SC, et al (2011) FOXQ1 Regulates Epithelial-Mesenchymal Transition in Human Cancers. Cancer Res 71: 3076–3086. 21346143 10.1158/0008-5472.CAN-10-2787

[99] Kaneda H, Arao T, Tanaka K, Tamura D, Aomatsu K, et al (2010) FOXQ1 is overexpressed in colorectal cancer and enhances tumorigenicity and tumor growth. Cancer Res 70: 2053–2063. 20145154 10.1158/0008-5472.CAN-09-2161

[100] Voelker D, Vess C, Tillmann M, Nagel R, Otto GW, et al (2007) Differential gene expression as a toxicant-sensitive endpoint in zebrafish embryos and larvae. Aquat Toxicol 81: 355–364. 17292976 10.1016/j.aquatox.2006.12.013

[101] Woltering DM (1984) The growth response in fish chronic and early life stage toxicity tests: A critical review. Aquat Toxicol 5: 1–21.

[102] Dennery PA (2007) Effects of oxidative stress on embryonic development. Birth Defects Res C Embryo Today 81: 155–162. 17963268 10.1002/bdrc.20098

[103] Rizzo AM, Adorni L, Montorfano G, Rossi F, Berra B (2007) Antioxidant metabolism of Xenopus laevis embryos during the first days of development. Comp Biochem Physiol B Biochem Mol Biol 146: 94–100. 17134930 10.1016/j.cbpb.2006.09.009

[104] Ishibashi M, Akazawa S, Sakamaki H, Matsumoto K, Yamasaki H, et al (1997) Oxygen-induced embryopathy and the significance of glutathione-dependent antioxidant system in the rat embryo during early organogenesis. Free Radic Biol Med 22: 447–454. 8981036 10.1016/s0891-5849(96)00338-3

[105] Manga P, Sheyn D, Yang F, Sarangarajan R, Boissy RE (2006) A role for tyrosinase-related protein 1 in 4-tert-butylphenol-induced toxicity in melanocytes: Implications for vitiligo. Am J Pathol 169: 1652–1662. 17071589 10.2353/ajpath.2006.050769 PMC1780195

[106] Jimenez-Cervantes C, Martinez-Esparza M, Perez C, Daum N, Solano F, et al (2001) Inhibition of melanogenesis in response to oxidative stress: transient downregulation of melanocyte differentiation markers and possible involvement of microphthalmia transcription factor. J Cell Sci 114: 2335–2344. 11493672 10.1242/jcs.114.12.2335

[107] Levy C, Khaled M, Fisher DE (2006) MITF: master regulator of melanocyte development and melanoma oncogene. Trends Mol Med 12: 406–414. 16899407 10.1016/j.molmed.2006.07.008

[108] Nasevicius A, Ekker SC (2000) Effective targeted gene ‘knockdown’ in zebrafish. Nat Genet 26: 216–220. 11017081 10.1038/79951

[109] Mellgren EM, Johnson SL (2004) A requirement for kit in embryonic zebrafish melanocyte differentiation is revealed by melanoblast delay. Dev Genes Evol 214: 493–502. 15300437 10.1007/s00427-004-0428-y

[110] Kingo K, Aunin E, Karelson M, Ratsep R, Silm H, et al (2008) Expressional changes in the intracellular melanogenesis pathways and their possible role the pathogenesis of vitiligo. J Dermatol Sci 52: 39–46. 18514490 10.1016/j.jdermsci.2008.03.013

[111] Yildirim M, Baysal V, Inaloz HS, Can M (2004) The role of oxidants and antioxidants in generalized vitiligo at tissue level. J Eur Acad Dermatol Venereol 18: 683–686. 15482295 10.1111/j.1468-3083.2004.01080.x

[112] Guan CP, Zhou MN, Xu AE, Kang KF, Liu JF, et al (2008) The susceptibility to vitiligo is associated with NF-E2-related factor2 (Nrf2) gene polymorphisms: a study on Chinese Han population. Exp Dermatol 17: 1059–1062. 18537816 10.1111/j.1600-0625.2008.00752.x

[113] Blechinger SR, Warren JT Jr, Kuwada JY, Krone PH (2002) Developmental toxicology of cadmium in living embryos of a stable transgenic zebrafish line. Environ Health Perspect 110: 1041–1046. 10.1289/ehp.021101041 PMC1241031 12361930

[114] Fratelli M, Goodwin LO, Orom UA, Lombardi S, Tonelli R, et al (2005) Gene expression profiling reveals a signaling role of glutathione in redox regulation. Proc Natl Acad Sci U S A 102: 13998–14003. 16172407 10.1073/pnas.0504398102 PMC1236550

[115] Zhang Y, Ahn YH, Benjamin IJ, Honda T, Hicks RJ, et al (2011) HSF1-Dependent Upregulation of Hsp70 by Sulfhydryl-Reactive Inducers of the KEAP1/NRF2/ARE Pathway. Chem Biol 18: 1355–1361. 22118669 10.1016/j.chembiol.2011.09.008 PMC3302153

[116] Goldstone JV, McArthur AG, Kubota A, Zanette J, Parente T, et al (2010) Identification and developmental expression of the full complement of Cytochrome P450 genes in Zebrafish. BMC Genomics 11: 643. 21087487 10.1186/1471-2164-11-643 PMC3012610

[117] Evans BR, Karchner SI, Franks DG, Hahn ME (2005) Duplicate aryl hydrocarbon receptor repressor genes (ahrr1 and ahrr2) in the zebrafish *Danio rerio*: Structure, function, evolution, and AHR-dependent regulation in vivo. Arch Biochem Biophys 441: 151–167. 16122694 10.1016/j.abb.2005.07.008

[118] Hayes KR, Bradfield CA (2005) Advances in toxicogenomics. Chem Res Toxicol 18: 403–414. 15777080 10.1021/tx0496690

[119] Churchill GA (2002) Fundamentals of experimental design for cDNA microarrays. Nat Genet 32 Suppl: 490–495. 12454643 10.1038/ng1031

[120] Novoradovskaya N, Whitfield ML, Basehore LS, Novoradovsky A, Pesich R, et al (2004) Universal Reference RNA as a standard for microarray experiments. BMC Genomics 5: 20. 15113400 10.1186/1471-2164-5-20 PMC394318

[121] Dobbin K, Shih JH, Simon R (2003) Questions and answers on design of dual-label microarrays for identifying differentially expressed genes. J Natl Cancer Inst 95: 1362–1369. 13130111 10.1093/jnci/djg049

[122] Quackenbush J (2002) Microarray data normalization and transformation. Nat Genet 32 Suppl: 496–501. 12454644 10.1038/ng1032

[123] Saeed AI, Sharov V, White J, Li J, Liang W, et al (2003) TM4: a free, open-source system for microarray data management and analysis. Biotechniques 34: 374–378. 12613259 10.2144/03342mt01

[124] Saeed AI, Bhagabati NK, Braisted JC, Liang W, Sharov V, et al (2006) TM4 microarray software suite. Methods Enzymol 411: 134–193. 16939790 10.1016/S0076-6879(06)11009-5

[125] Benjamini Y, Hochberg Y (1995) Controlling the false discovery rate: a practical and powerful approach to multiple testing. J R Stat Soc Ser B 57: 289–3000.

[126] Korn EL, Troendle JF, McShane LM, Simon R (2004) Controlling the number of false discoveries: application to high-dimensional genomic data. J Stat Plan Infer 124: 379–398.

[127] Eisen MB, Spellman PT, Brown PO, Botstein D (1998) Cluster analysis and display of genome-wide expression patterns. Proc Natl Acad Sci U S A 95: 14863–14868. 9843981 10.1073/pnas.95.25.14863 PMC24541

[128] Howe K, Clark MD, Torroja CF, Torrance J, Berthelot C, et al (2013) The zebrafish reference genome sequence and its relationship to the human genome. Nature 496: 498–503. 23594743 10.1038/nature12111 PMC3703927

[129] Pruess M, Kersey P, Apweiler R (2005) The Integr8 project–a resource for genomic and proteomic data. In Silico Biol 5: 179–185. 15972013

[130] Bailey TL, Gribskov M (1998) Combining evidence using p-values: application to sequence homology searches. Bioinformatics 14: 48–54. 9520501 10.1093/bioinformatics/14.1.48

[131] Storey JD, Tibshirani R (2003) Statistical significance for genomewide studies. Proc Natl Acad Sci U S A 100: 9440–9445. 12883005 10.1073/pnas.1530509100 PMC170937

[132] Wang X, Tomso DJ, Chorley BN, Cho HY, Cheung VG, et al (2007) Identification of polymorphic antioxidant response elements in the human genome. Hum Mol Genet 16: 1188–1200. 17409198 10.1093/hmg/ddm066 PMC2805149

[133] Sun YV, Boverhof DR, Burgoon LD, Fielden MR, Zacharewski TR (2004) Comparative analysis of dioxin response elements in human, mouse and rat genomic sequences. Nucleic Acids Res 32: 4512–4523. 15328365 10.1093/nar/gkh782 PMC516056

[134] Stender JD, Kim K, Charn TH, Komm B, Chang KC, et al (2010) Genome-wide analysis of estrogen receptor alpha DNA binding and tethering mechanisms identifies Runx1 as a novel tethering factor in receptor-mediated transcriptional activation. Molecular and Cellular Biology 30: 3943–3955. 20547749 10.1128/MCB.00118-10 PMC2916448

[135] Malhotra D, Portales-Casamar E, Singh A, Srivastava S, Arenillas D, et al (2010) Global mapping of binding sites for Nrf2 identifies novel targets in cell survival response through ChIP-Seq profiling and network analysis. Nucleic Acids Res 38: 5718–5734. 20460467 10.1093/nar/gkq212 PMC2943601

[136] Cui JY, Gunewardena SS, Rockwell CE, Klaassen CD (2010) ChIPing the cistrome of PXR in mouse liver. Nucleic Acids Res 38: 7943–7963. 20693526 10.1093/nar/gkq654 PMC3001051

[137] Wasserman WW, Sandelin A (2004) Applied bioinformatics for the identification of regulatory elements. Nat Rev Genet 5: 276–287. 15131651 10.1038/nrg1315

