# Supplementary material for: The Transcriptional Response to Oxidative Stress during Vertebrate Development: Effects of tert-Butylhydroquinone and 2,3,7,8-Tetrachlorodibenzo-p-Dioxin
Source: PLoS One. 2014 Nov 17;9(11):e113158. doi: 10.1371/journal.pone.0113158 (PMC4234671; doi:10.1371/journal.pone.0113158)
Supplement: Table S1 — Primers for Real-time RT-PCR. (DOCX) [file pone.0113158.s001.docx]

**Table S1. Primers for Real-time RT-PCR**

| ***gene*** | **qPCR Primer sequence** |
| --- | --- |
| *gstp1* | CGACTTGAAAGCCACCTGTGTC |
|  | CTGTCGTTTTTGCCATATGCAGC |
| *gcsh* | GGAGGTGTTTCCAGAGGATG |
|  | AAGCCTGGAAGGTCACCTG |
| *nqo1* | TTCAGTACCCACTCTACTGG |
|  | GCATGGCCCTCTTATTCTTG |
| *sod1* | CGTCTATTTCAATCAAGAGGGTG |
|  | GATGCAGCCGTTTGTGTTGTC |
| *gadd45* | CAGCACTGAGAGAAAAATGG |
|  | TCATGAGTTGTGCAGACTCG |
| *foxq1b* | CTAATGCTGTCAGCTGCTCC |
|  | CCAAACCCTCAGAACTGACC |
| *mitfa* | GATGGCTTTCCAGTAGAAGC |
|  | GCCTTCTTCGTTCAATGAGG |
| *atf3* | TCCAGAACAAACGCATGTCC |
|  | TCCTCTGGGACAGCCTCTCG |
| *opn1lw1* | CGTTCTTCGCCTGCTTTGCAGC |
|  | TGATGCATACGCGGAACTGTCG |
| *cyp1a* | GCATTACGATACGTTCGATAAGGAC |
|  | GCTCCGAATAGGTCATTGACGAT |
| *nrf2a* | GAGCGGGAGAAATCACACAGAATG |
|  | CAGGAGCTGCATGCACTCATCG |
| *hsp70* | GAAGACGGCATCTTTGAGGTGA |
|  | GGGCCCTCTTGTTCTGACTGAT |
