# Supplementary material for: The Transcriptional Response to Oxidative Stress during Vertebrate Development: Effects of tert-Butylhydroquinone and 2,3,7,8-Tetrachlorodibenzo-p-Dioxin
Source: PLoS One. 2014 Nov 17;9(11):e113158. doi: 10.1371/journal.pone.0113158 (PMC4234671; doi:10.1371/journal.pone.0113158)
Supplement: Table S3 — Enrichment of Gene Ontology (GO) terms for Ensembl annotated probes with significantly different abundance between TCDD, tBHQ, and DMSO treated eleutheroembryos. (DOC) [file pone.0113158.s003.doc]

**Table S3. Enrichment of Gene Ontology (GO) terms for Ensembl annotated probes with significantly different abundance between TCDD, tBHQ, and DMSO treated embryos (p < 0.05, ANOVA with 5% FDR correction).**

Groupings represent the clusters presented in Figure 3. Enriched GO terms were detected using the FatiGO+ algorithm for each cluster of probes relative to the background set of probes (Fisher's exact test, p < 0.01). Levels of GO terms range from broad (level 3) to specific (level 9). Frequencies of probes assigned each GO term are presented for both significant and background sets of probes, with percentages calculated based on the total number of probes involving GO terms at each GO level in question). FatiGO+ uses Nested Inclusive Analysis (NIA), which takes into account the tree-like structure of the GO hierarchy such that only the most specific GO level with significance is reported. For example, while 36 probes in the table reflected enrichment of the GO term “regulation of transcription” for tBHQ down-regulation, another 37 could only be assigned to the more general GO term “transcription” based on Ensembl GO assignments.

| **GO Term** | **GO Level** | **Significant Probes** | **Background Probes** |
| --- | --- | --- | --- |
|  |  |  |  |
| *tBHQ Up-Regulated* |  |  |  |
| catabolic process | Biological Process, Level 3 | 10 (9.71%) | 77 (3.36%) |
| response to temperature stimulus | Biological Process, Level 4 | 4 (3.96%) | 2 (0.09%) |
| dephosphorylation | Biological Process, Level 6 | 6 (6.98%) | 31 (1.72%) |
| negative regulation of signal transduction | Biological Process, Level 6 | 3 (3.49%) | 3 (0.17%) |
| glutathione metabolic process | Biological Process, Level 6 | 2 (2.33%) | 0 (0.00%) |
| glutamine family amino acid catabolic process | Biological Process, Level 8 | 2 (3.57%) | 1 (0.11%) |
| membrane-bound organelle | Cellular Component, Level 3 | 40 (62.50%) | 745 (43.06%) |
| organelle part | Cellular Component, Level 3 | 17 (26.56%) | 235 (13.58%) |
| intracellular membrane-bound organelle | Cellular Component, Level 7 | 40 (80.00%) | 745 (57.84%) |
| protein dimerization activity | Molecular Function, Level 4 | 7 (7.29%) | 20 (0.89%) |
| DNA photolyase activity | Molecular Function, Level 5 | 3 (3.41%) | 4 (0.20%) |
| phosphoric ester hydrolase activity | Molecular Function, Level 5 | 7 (7.95%) | 49 (2.40%) |
| phosphoric monoester hydrolase activity | Molecular Function, Level 6 | 7 (10.94%) | 45 (3.05%) |
| phosphoprotein phosphatase activity | Molecular Function, Level 7 | 6 (24.00%) | 33 (5.54%) |
| protein tryosine/serine/threonine phosphatase activity | Molecular Function, Level 8 | 6 (46.15%) | 16 (5.67%) |
| protein tyrosine phosphatase activity | Molecular Function, Level 8 | 4 (30.77%) | 9 (3.19%) |
| MAP kinase phosphatase activity | Molecular Function, Level 9 | 3 (50.00%) | 0 (0.00%) |
|  |  |  |  |
| *tBHQ Down-Regulated* |  |  |  |
| regulation of biological process | Biological Process, Level 3 | 44 (34.65%) | 517 (22.64%) |
| nucleobase, nucleoside, nucleotide and nucleic acid metabolic process | Biological Process, Level 4 | 53 (43.09%) | 626 (28.05%) |
| regulation of cellular process | Biological Process, Level 4 | 43 (34.96%) | 482 (21.59%) |
| regulation of metabolic process | Biological Process, Level 4 | 37 (30.08%) | 401 (17.97%) |
| regulation of cellular metabolic process | Biological Process, Level 5 | 37 (33.04%) | 394 (19.29%) |
| negative regulation of cellular process | Biological Process, Level 5 | 7 (6.25%) | 37 (1.81%) |
| RNA metabolic process | Biological Process, Level 5 | 38 (33.93%) | 421 (20.61%) |
| transcription | Biological Process, Level 5 | 37 (33.04%) | 397 (19.43%) |
| regulation of nucleobase, nucleoside, nucleotide and nucleic acid metabolic process | Biological Process, Level 6 | 36 (35.29%) | 384 (21.29%) |
| RNA biosynthetic process | Biological Process, Level 6 | 32 (31.37%) | 336 (18.63%) |
| regulation of transcription | Biological Process, Level 7 | 36 (43.37%) | 382 (28.98%) |
| membrane-bound organelle | Cellular Component, Level 3 | 64 (57.66%) | 742 (43.14%) |
| intracellular organelle | Cellular Component, Level 6 | 69 (77.53%) | 877 (62.33%) |
| cytoplasm | Cellular Component, Level 6 | 12 (13.48%) | 369 (26.23%) |
| intracellular membrane-bound organelle | Cellular Component, Level 7 | 64 (74.42%) | 742 (57.74%) |
| cytoplasmic part | Cellular Component, Level 7 | 7 (8.14%) | 285 (22.18%) |
| nucleus | Cellular Component, Level 8 | 59 (78.67%) | 571 (55.93%) |
| nuclear part | Cellular Component, Level 9 | 10 (52.63%) | 56 (17.02%) |
| nucleic acid binding | Molecular Function, Level 3 | 50 (35.21%) | 643 (24.53%) |
| cytoskeletal protein binding | Molecular Function, Level 4 | 6 (4.84%) | 27 (1.20%) |
| purine nucleotide binding | Molecular Function, Level 4 | 10 (8.06%) | 415 (18.52%) |
| adenyl nucleotide binding | Molecular Function, Level 5 | 5 (4.63%) | 301 (14.81%) |
| cysteine-type peptidase activity | Molecular Function, Level 5 | 7 (6.48%) | 29 (1.43%) |
| sequence-specific DNA binding | Molecular Function, Level 5 | 18 (16.67%) | 177 (8.71%) |
| cysteine-type endopeptidase activity | Molecular Function, Level 6 | 7 (10.77%) | 26 (1.77%) |
| calpain activity | Molecular Function, Level 7 | 2 (8.00%) | 2 (0.34%) |
| caspase activity | Molecular Function, Level 7 | 3 (12.00%) | 5 (0.84%) |
|  |  |  |  |
| *TCDD Up-Regulated* |  |  |  |
| regulation of signal transduction | Biological Process, Level 5 | 2 (18.18%) | 17 (0.81%) |
| ameboidal cell migration | Biological Process, Level 6 | 1 (10.00%) | 0 (0.00%) |
| cell projection morphogenesis | Biological Process, Level 6 | 2 (20.00%) | 21 (1.14%) |
| smoothened signaling pathway | Biological Process, Level 6 | 1 (10.00%) | 0 (0.00%) |
| vesicle localization | Biological Process, Level 6 | 1 (10.00%) | 0 (0.00%) |
| cell projection organization and biogenesis | Biological Process, Level 7 | 2 (22.22%) | 21 (1.56%) |
| dicarboxylic acid transport | Biological Process, Level 7 | 1 (11.11%) | 0 (0.00%) |
| establishment of vesicle localization | Biological Process, Level 7 | 1 (11.11%) | 0 (0.00%) |
| pigment granule localization | Biological Process, Level 7 | 1 (11.11%) | 0 (0.00%) |
| regulation of smoothened signaling pathway | Biological Process, Level 7 | 1 (11.11%) | 0 (0.00%) |
| establishment of pigment granule localization | Biological Process, Level 8 | 1 (16.67%) | 0 (0.00%) |
| pigment granule aggregation in cell center | Biological Process, Level 9 | 1 (33.33%) | 0 (0.00%) |
| tetrapyrrole binding | Molecular Function, Level 3 | 2 (22.22%) | 37 (1.38%) |
| heme binding | Molecular Function, Level 4 | 2 (22.22%) | 37 (1.61%) |
| kinase inhibitor activity | Molecular Function, Level 4 | 1 (11.11%) | 1 (0.04%) |
| oxidoreductase activity, acting on paired donors, with incorporation or reduction of molecular oxygen | Molecular Function, Level 4 | 2 (22.22%) | 36 (1.57%) |
| dicarboxylic acid transporter activity | Molecular Function, Level 5 | 1 (11.11%) | 0 (0.00%) |
| protein kinase inhibitor activity | Molecular Function, Level 5 | 1 (11.11%) | 1 (0.05%) |
| retinoic acid 4-hydroxylase activity | Molecular Function, Level 5 | 1 (11.11%) | 0 (0.00%) |
| cyclin-dependent protein kinase inhibitor activity | Molecular Function, Level 6 | 1 (14.29%) | 1 (0.07%) |
| anion:cation symporter activity | Molecular Function, Level 7 | 1 (33.33%) | 0 (0.00%) |
|  |  |  |  |
| *TCDD Down-Regulated* |  |  |  |
| folic acid binding | Molecular Function, Level 4 | 1 (11.11%) | 0 (0.00%) |
| voltage-gated ion channel activity | Molecular Function, Level 6 | 2 (40.00%) | 22 (1.47%) |
