# Supplementary material for: The Transcriptional Response to Oxidative Stress during Vertebrate Development: Effects of tert-Butylhydroquinone and 2,3,7,8-Tetrachlorodibenzo-p-Dioxin
Source: PLoS One. 2014 Nov 17;9(11):e113158. doi: 10.1371/journal.pone.0113158 (PMC4234671; doi:10.1371/journal.pone.0113158)
Supplement: Table S5 — Comparison of altered gene expression as measured by microarray and qRT-PCR. (DOC) [file pone.0113158.s005.doc]

**Table S5. Comparison of altered gene expression as measured by microarray and qRT-PCR.**

Data are from 4-dpf embryos treated with tBHQ or TCDD. Arrows indicate statistically significant changes as compared to gene expression in DMSO-exposed control embryos. Dashes indicate no change from controls. Numbers in parentheses indicate the ratio of gene expression in tBHQ- or TCDD-exposed embryos to that in DMSO-exposed embryos.

|  | **tBHQ** | | **TCDD** | |
| --- | --- | --- | --- | --- |
| **Gene** | **array** | **qRT-PCR** | **array** | **qRT-PCR** |
| *gstp1* | ↑ (2.9)^a^ | ↑ (4.3)^a^ | -- | -- |
| *gclc* | ↑ (2.2) | ↑ (3.4) | -- | -- |
| *sod1* | -- | -- | -- | -- |
| *nqo1* | -- | -- | -- | -- |
| *cyp1a* | -- | -- | ↑ (72) | ↑ (358) |
| *nrf2a* | -- | -- | -- | -- |
| *hsp70* | ↑ (52) | ↑ (153) | ↑ (3.9) | ↑ (8.7) |
| *gadd45b* | ↑ (10) | ↑ (14) | -- | -- |
| *atf3* | ↑ (19) | ↑ (42) | -- | -- |
| *mitfa* | ↓ (0.35) | ↓ (0.19) | -- | -- |
| *opsin* | ↓ (0.17) | ↓ (0.16) | -- | -- |
| *foxq1b* | ↓ (0.43) | -- (1.0) | ↑ (3.5) | ↑ (5.5) |

^a^ Change was not statistically significant.
